# The Genomic Contributions of Avian H1N1 Influenza A Viruses to the Evolution of Mammalian Strains

**DOI:** 10.1371/journal.pone.0133795

**Published:** 2015-07-24

**Authors:** Zeynep A. Koçer, Robert Carter, Gang Wu, Jinghui Zhang, Robert G. Webster

**Affiliations:** 1 Department of Infectious Diseases, Division of Virology, St. Jude Children’s Research Hospital, Memphis, Tennessee, United States of America; 2 Department of Computational Biology, St. Jude Children’s Research Hospital, Memphis, Tennessee, United States of America; 3 Department of Biological Sciences, Faculty of Science, King Abdulaziz University, Jeddah, Saudi Arabia; University of Edinburgh, UNITED KINGDOM

## Abstract

Among the influenza A viruses (IAVs) in wild aquatic birds, only H1, H2, and H3 subtypes have caused epidemics in humans. H1N1 viruses of avian origin have also caused 3 of 5 pandemics. To understand the reappearance of H1N1 in the context of pandemic emergence, we investigated whether avian H1N1 IAVs have contributed to the evolution of human, swine, and 2009 pandemic H1N1 IAVs. On the basis of phylogenetic analysis, we concluded that the polymerase gene segments (especially PB2 and PA) circulating in North American avian H1N1 IAVs have been reintroduced to swine multiple times, resulting in different lineages that led to the emergence of the 2009 pandemic H1N1 IAVs. Moreover, the similar topologies of hemagglutinin and nucleoprotein and neuraminidase and matrix gene segments suggest that each surface glycoprotein coevolved with an internal gene segment within the H1N1 subtype. The genotype of avian H1N1 IAVs of Charadriiformes origin isolated in 2009 differs from that of avian H1N1 IAVs of Anseriformes origin. When the antigenic sites in the hemagglutinin of all 31 North American avian H1N1 IAVs were considered, 60%-80% of the amino acids at the antigenic sites were identical to those in 1918 and/or 2009 pandemic H1N1 viruses. Thus, although the pathogenicity of avian H1N1 IAVs could not be inferred from the phylogeny due to the small dataset, the evolutionary process within the H1N1 IAV subtype suggests that the circulation of H1N1 IAVs in wild birds poses a continuous threat for future influenza pandemics in humans.

## Introduction

Influenza A viruses (IAVs) belong to the *Orthomyxoviridae* family of negative-sense, single-stranded RNA viruses. Their genome is composed of 8 segments coding as many as 12 proteins (PB2, PB1, PB1-F2, PA, PA-X, HA, NP, NA, M1, M2, NS1, and NEP) in avian IAVs [1–4].

The major reservoir for IAVs is wild aquatic and migratory birds [5]. Currently, there are 18 hemagglutinin (HA) and 11 neuraminidase (NA) subtypes of IAVs, of which 16 HA and 9 NA subtypes are circulating in birds and 2 HA subtypes are found in bats [5–9]. Although most IAV subtypes are detected in the birds of Anseriformes origin (e.g., mallards, ducks), H13 and H16 subtypes are prevalent in those of Charadriiformes origin (shorebirds, gulls) [10].

The 1918 H1N1 pandemic virus is believed to have originated from an avian population and then entered swine and human populations around the same time. Subsequently, it became established in swine (classical swine), caused a pandemic in humans, and then became established in the human population [11,12]. In 1979, Eurasian avian—like H1N1 IAVs entered the Eurasian swine population and have been co-circulating with the classical swine virus in Eurasia ever since [13]. The 1957 and 1968 pandemics caused by H2N2 and H3N2 subtypes, respectively, replaced the H1N1 virus in the human population until its re-appearance in 1977. Since then, H1N1 virus has been circulating in humans, along with seasonal H3N2 virus. Yet, the novel H1N1 virus emerged in 2009 with a unique gene constellation of avian, swine, and human-origin segments [14,15] and caused a pandemic, despite the existing immune response against human seasonal H1N1.

Although we know that all IAVs originated from avian sources and that the 1918 H1N1 pandemic IAV had a purely avian origin [16], our knowledge regarding the degree to which the avian H1N1 IAVs have contributed to the mammalian IAV gene pool over the last century is limited. Nor do we know how readily avian H1N1 IAVs might contribute to mammalian IAVs in the future. An understanding of this frequency is important for public health, because we have previously demonstrated that some avian H1N1 IAVs can cause disease in mammalian models and some can transmit by direct-contact and/or respiratory-droplet in ferrets [17].

The current study investigates the evolutionary history of H1N1-subtype IAVs isolated from avian, swine, or human hosts around the globe to assess the potential risks posed to humans by wild birds. This was accomplished via a genome-scale phylogenetic assessment of all 8 segments of avian, swine, and human H1N1 IAV isolates from Eurasian and North American strains isolated between 1976 and 2012. We investigated the continuing contribution of avian H1N1 IAVs to the evolution of swine, human, and the 2009 pandemic H1N1 IAVs. To understand the importance of H1N1 IAV emergence in birds of Charadriiformes origin in 2009, we also address the genetic and antigenic relatedness of these viruses to the H1N1 IAVs of Anseriformes origin and to the past pandemic and seasonal human H1N1 IAVs.

## Materials and Methods

### Nucleotide sequences

The GenBank accession numbers for the full genomes of H1N1 IAVs of avian, swine, and human origin (n = 354 taxa) are presented in S1 Table. The previously published pathogenicity indexes of the 31 North American avian H1N1 IAVs in the St. Jude Children’s Research Hospital (St. Jude) influenza repository are also included in that table [17].

### Phylogenetic analyses

To acquire a representative and nonredundant set of H1N1 sequences, we downloaded an initial set of 24,550 publicly available gene segments (3068 full genome) from H1N1 IAVs of avian, swine, and human origins [North American avian (n = 98); swine (n = 976); human (n = 5392); Eurasian avian (n = 240);, swine (n = 2072); human (n = 2123), and 2009 pandemic (n = 13649) viruses] from the Influenza Virus Resource on NCBI in April 2013 (http://www.ncbi.nlm.nih.gov/genomes/FLU/FLU.html). The genomes were clustered at the 99% identity level and a single representative from each cluster was retained for further analysis. Clustering was performed at the whole-genome level by obtaining the coding sequence of each segment of a strain, concatenating them, and then clustering the resulting genomes using the cluster_fast method of Usearch [18]. For segments with multiple gene products, all intervening sequences between the terminal 5' start codon and the terminal 3' stop codon were considered coding sequences. There were 354 strains [North American avian (n = 58), North American swine (n = 62), North American human (n = 45), Eurasian avian (n = 6), Eurasian swine (n = 111), Eurasian human (n = 31), and 2009 pandemic H1N1 (n = 10)] with sequences for each gene following the clustering procedure, including the 31 North American H1N1 IAVs. The PA gene of A/mallard/MN/AI07-3136/2007 and the M gene of A/mallard/MN/AI07-3140/2007 were not included in the phylogenetic analyses due to the high number of ambiguous positions detected in the consensus sequence.

BEAST software was used to estimate phylogenetic trees separately for each gene product [19]. The coding sequences of segments 1 through 6 were partitioned into 2 groups, each having its own relative rate, rate distribution, and proportion of invariable sites to account for the nature of the genetic code [20]. Group 1 consisted of codon positions 1 and 2, whereas group 2 consisted of codon position 3. The coding sequences of segments encoding NS1 and NEP on one hand and M1 and M2 on the other were treated differently than the other 6 segments due to the presence of overlapping transcripts in different reading frames. For both of these segments, codons were extracted from either transcript only if they were part of a single transcript. In both segments, this resulted in coding sequences that were hybrids of each transcript. It was assumed that rates along lineages follow an uncorrelated relaxed molecular clock [21] and evolve according to the HKY substitution model. Default or noninformative priors were used in most cases including a coalescent constant population prior, with the following exceptions. The prior mean rate across branches (ucld.mean) was assumed to follow a broad-normal distribution, with a mean of 0.003 and a standard deviation of 0.01. This mean lies between the estimated number of synonymous and nonsynonymous substitutions per year in IAV sequences [22]. The prior probability of the standard deviation of the distribution of branch rates was assumed to follow an exponential distribution with a mean of 0.001. Fifty million generations of MCMC were run for each segment and used to generate the maximum clade credibility trees using TreeAnnotator software [19].

## Results

To understand the continuing contribution of avian H1N1 IAVs to the evolution of swine, human, and 2009 pandemic H1N1 IAVs, we performed a comprehensive phylogenetic analysis of the H1N1 subtype from various hosts and geographic regions. We included 354 representative taxa from public databases comprising isolates from avian, swine, and human hosts in Eurasia and North America, as well as the 31 North American avian H1N1 IAVs from the influenza repository at St. Jude that were previously sequenced [23,24].

### Recent introduction of avian polymerase gene segments to mammalian H1N1 IAVs

#### PB2 gene segment

**Fig 1:**
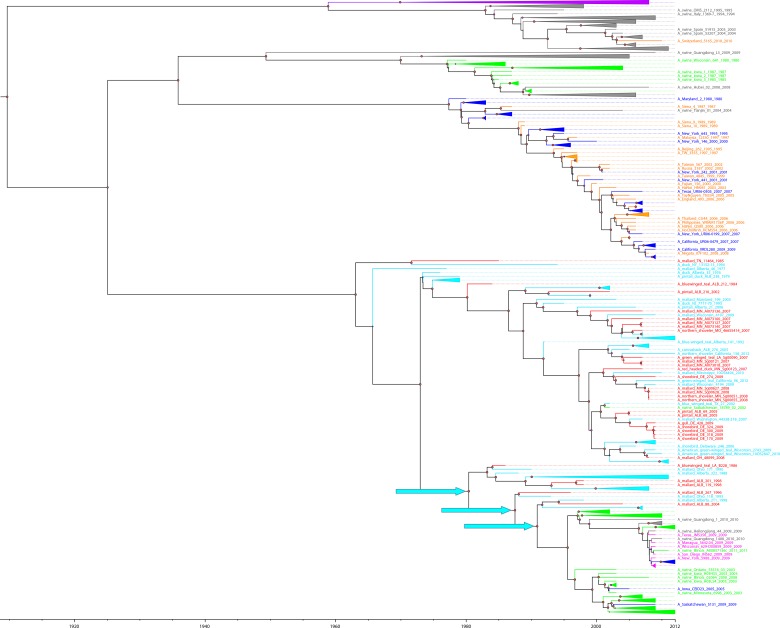
Evolutionary relatedness of PB2 gene segments of H1N1 IAVs of avian, swine, and human origins isolated from North America and Eurasia. The taxa are colored based on their host and geography: red-North American avian isolates from the St. Jude repository, light blue-North American avian isolates, purple-Eurasian avian isolates, green- swine isolates from North America, grey- swine isolates from Eurasia (both Eurasian avian-like swine and Eurasian classical swine), dark blue-human isolates from North America; orange- human isolates from Eurasia, and pink- 2009 pandemic isolates. The nodes with spillover avian H1N1 IAVs are denoted with light blue arrows.

**Fig 2:**
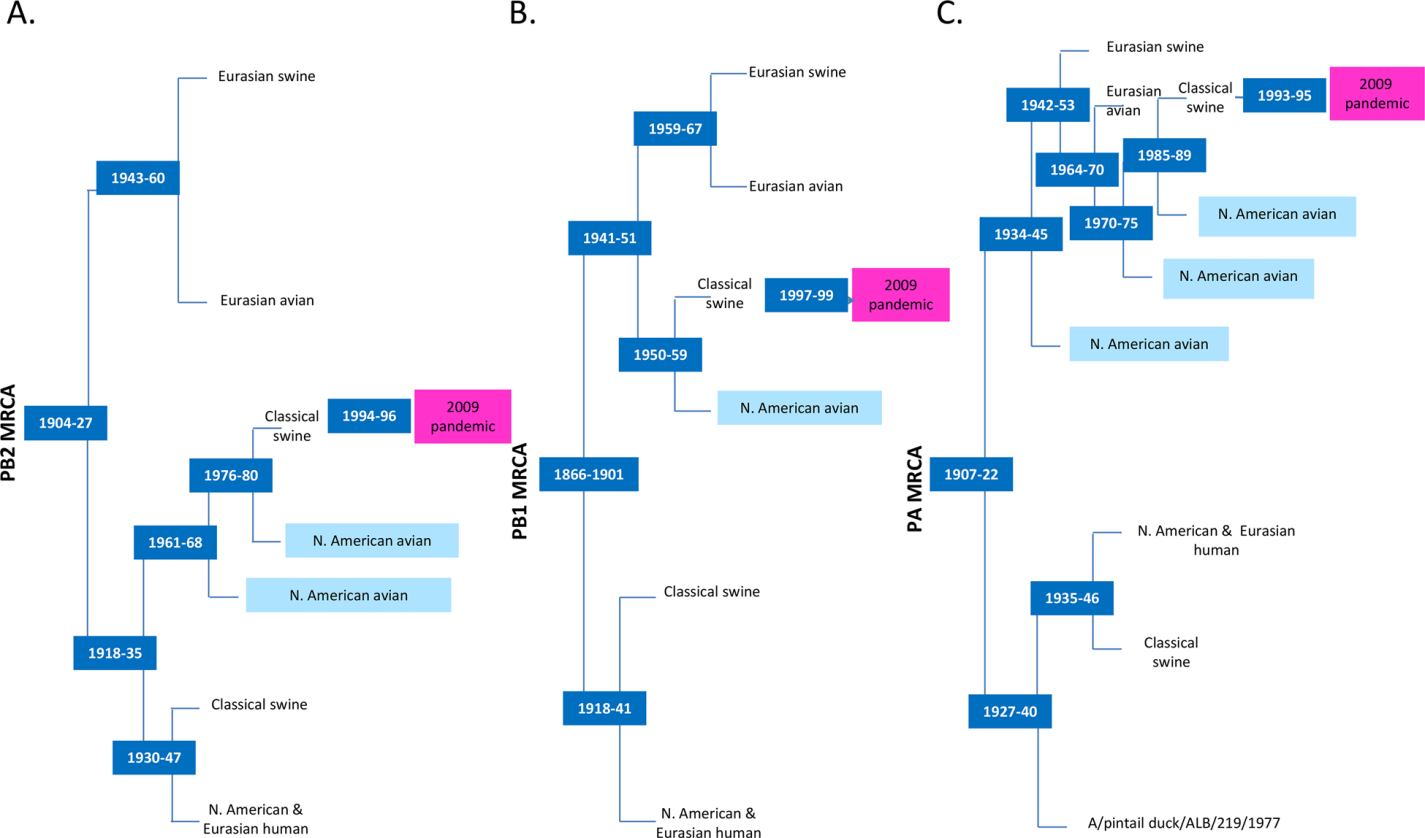
Schematic of the evolutionary history among PB2, PB1 and PA segments of H1N1 IAVs. The schematic view is based on the phylogenetic trees of avian, swine, and human origin H1N1 IAVs and the most recent common ancestor (MRCA) for (A) PB2 gene segments, (B) PB1 gene segments, and (C) PA gene segments. North American avian (light blue) and 2009 pandemic H1N1 viruses (pink) are highlighted.

To understand the contribution of polymerase gene segments (PB2, PB1, and PA) of H1N1 IAVs from birds to swine and humans, we investigated the evolutionary history of the full-length polymerase gene segments of H1N1 IAVs from different host origins. The PB2 gene segments of H1N1 IAVs shared the most recent common ancestor (MRCA) around 1904–1927. The ancestral PB2 gene segment diverged into 2 lineages forming a strictly Eurasian clade consisting of Eurasian avian and Eurasian avian—like swine H1N1 IAVs and a heterogeneous clade composed of classical swine, human, and North American avian H1N1 IAVs (Figs 1 and 2A). With the exception of a single swine-to-human event (A/Switzerland/5165/2010), the root node of the tree is the MRCA of avian and swine H1N1 IAVs but not of human isolates. Furthermore, the geographic origin of the avian sequences is coincident with the 2 major lineages, suggesting little-to-no intercontinental exchange of avian PB2 gene segments. In contrast, classical swine isolates from both geographic regions are present in both lineages, indicating widespread intercontinental exchange.

The MRCA of the PB2 gene segments from human H1N1 IAVs is within the heterogeneous clade. The human H1N1 IAVs are most closely related to classical swine isolates, because both are found within the same clade that diverged in the early 1920s. The PB2 gene segment of the 2009 pandemic IAVs was acquired from the classical swine lineage within the heterogeneous clade that was recently introduced from the avian to the swine population (Fig 1). A group of North American avian H1N1 IAVs, including 5 of 31 viruses from our repository (A/blue winged teal/LA/B228/1986, A/mallard/ALB/119/1998, A/mallard/ALB/201/1998, A/mallard/ALB/267/1996, and A/mallard/ALB/88/2004) shared the MRCA with North American classical swine and the 2009 pandemic viruses until the 1980s–1990s (Figs 1 and 2A). Eleven North American avian H1N1 IAVs, including 3 from our repository (A/blue winged teal/LA/B228/1986, A/mallard/ALB/119/1998, and A/mallard/ALB/201/1998), shared a MRCA with North American swine and the 2009 pandemic viruses until the late 1970s to early 1980s. Five North American avian H1N1 IAVs, including A/mallard/ALB/267/1996 from our repository, diverged from the MRCA with North American swine and the 2009 pandemic viruses in 1988. Among all North American avian H1N1 IAVs, a group of avian H1N1 IAVs, including only 1 virus from our repository (A/mallard/ALB/88/2004) shared the MRCA with North American swine and the 2009 pandemic viruses in 1991.

#### PB1 gene segment

Unlike the PB2 segment, the basal-most split in the PB1 tree is largely coincident with host origin (Fig 2B and S1 Fig). The 2 resulting lineages diverged from their MRCA between 1866 and 1901. Isolates from both human and swine, as well as both geographic regions are present in each basal clade, but avian isolates are restricted to only one clade. One lineage led to the PB1 gene segments circulating in classical swine and human H1N1 IAVs; the other led to those circulating in Eurasian avian, Eurasian avian—like swine, North American avian, North American classical swine, and eventually the 2009 pandemic H1N1 viruses. From the latter clade, PB1 gene segments of Eurasian avian and avian—like swine H1N1 IAVs diverged from North American avian and classical swine viruses during 1941–1951. North American avian and classical swine H1N1 IAVs diverged during 1950–1959. A group of North American classical swine H1N1 IAVs shared a MRCA with an avian H1N1 virus (A/duck/Alberta/35/1976) around 1967, some of which led to the emergence of PB1 segments of the 2009 pandemic H1N1 (S1 Fig). Unlike PB2 gene segments, PB1 gene segments from Eurasian avian and avian—like swine H1N1 IAVs diverged from the MRCA with North American avian H1N1 IAVs more recently (1948). With the exception of some putative isolated reassortment events, the evolutionary relations were similar between the 2 segments, including the close relations between swine and human PB1 sequences at different times.

#### PA gene segment

**Fig 3:**
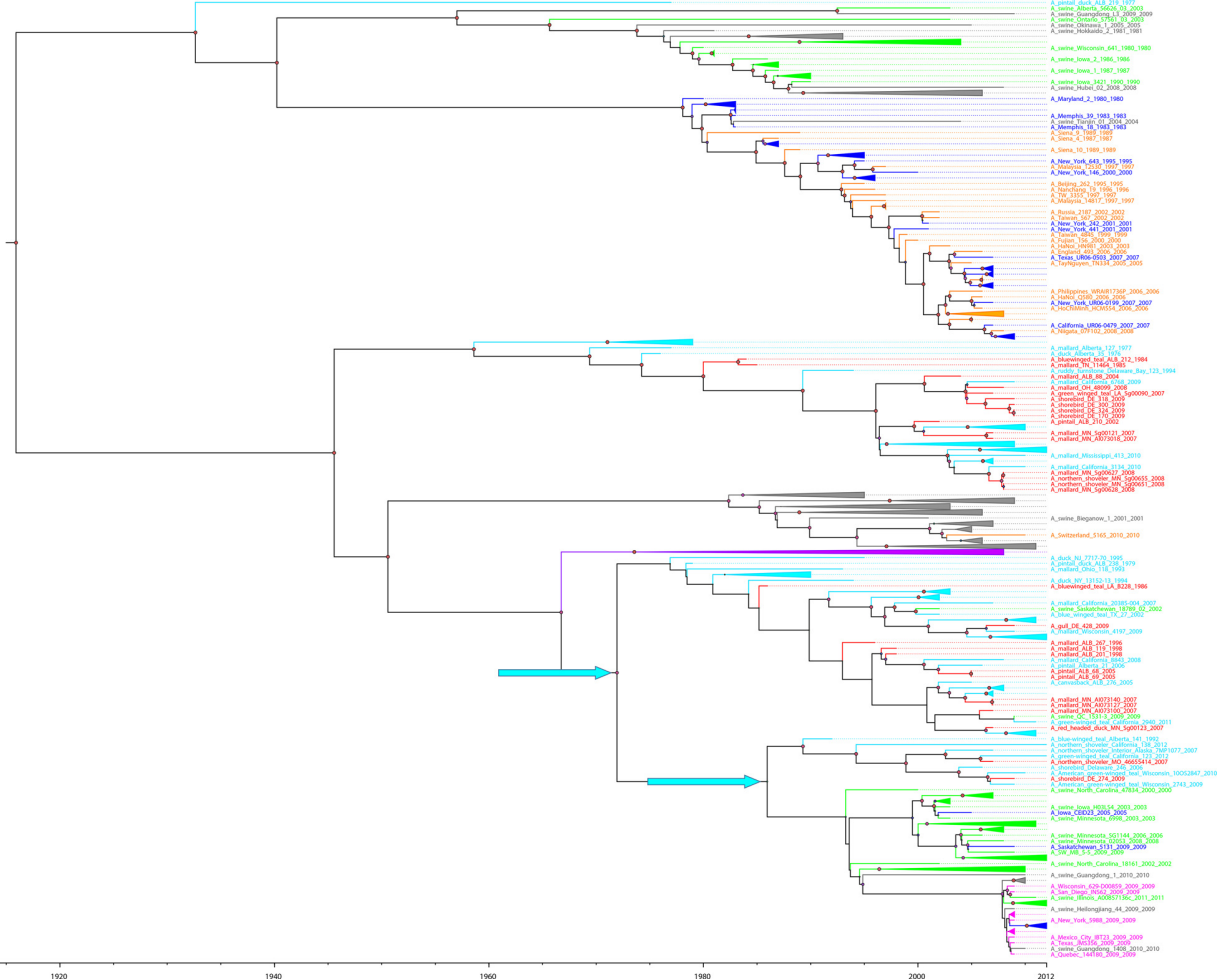
Evolutionary relatedness of PA gene segments of H1N1 IAVs of avian, swine and human origins isolated from North America and Eurasia. The taxa are colored based on their host and geography: red-North American avian isolates from the St. Jude repository, light blue-North American avian isolates, purple-Eurasian avian isolates, green- swine isolates from North America, grey- swine isolates from Eurasia (both Eurasian avian-like swine and Eurasian classical swine), dark blue-human isolates from North America; orange- human isolates from Eurasia, and pink- 2009 pandemic isolates. The nodes with spillover avian H1N1 IAVs are denoted with light blue arrows.

The ancestral PA gene segment circulated around 1907–1922. Similar to the divergence in the PB1 segment, the most basal divergence in the phylogenetic tree of the PA gene segment led to clades that were distinguished by host instead of geographic origin. Most likely introduced from an avian source in the 1930s, one clade led to the descendants of PA gene segments circulating in North American and Eurasian human and classical swine H1N1 IAVs. The other clade gave rise to North American and Eurasian avian H1N1 IAVs, as well as to some PA gene segments circulating in North American classical swine and Eurasian avian—like swine H1N1 viruses (Figs 2C and 3). In this latter clade, a group of North American avian H1N1 IAVs descended from the MRCA between 1934 and 1945. After the divergence of Eurasian avian—like swine H1N1 IAVs between 1942 and 1953, the PA gene segments circulating in Eurasian avian H1N1 IAVs formed a monophyletic clade. Until the late 1980s, the avian PA gene segments were introduced to swine populations multiple times and eventually seeded the PA gene segment of the 2009 pandemic virus (Figs 2C and 3).

Overall, although the topologies of the polymerase gene segments are largely congruent, the main topological differences are caused by the divergence time of Eurasian avian and avian—like swine H1N1 IAVs from different ancestral sources in the evolution of each polymerase gene segments (Fig 2). Historically, swine and human H1N1 IAV polymerase gene segments have frequently been exchanged between distant geographic regions, whereas little exchange of avian polymerase segments has occurred. Swine sequences appear to be a recurrent source of polymerase gene segments for human H1N1 IAVs. Earlier North American classical swine polymerase gene segments led to the evolution of human H1N1 IAVs; however, the recent introduction of polymerase gene segments from avian to classical swine H1N1 IAVs resulted in the emergence of the 2009 pandemic H1N1 viruses.

### Coevolution of the HA-NP and NA-M gene segments

We examined the contribution of surface glycoproteins (HA and NA) and internal genes [i.e., nucleoprotein (NP) and matrix (M) genes] of avian H1N1 IAVs to the evolution of swine, human, and pandemic H1N1 IAVs.

**Fig 4:**
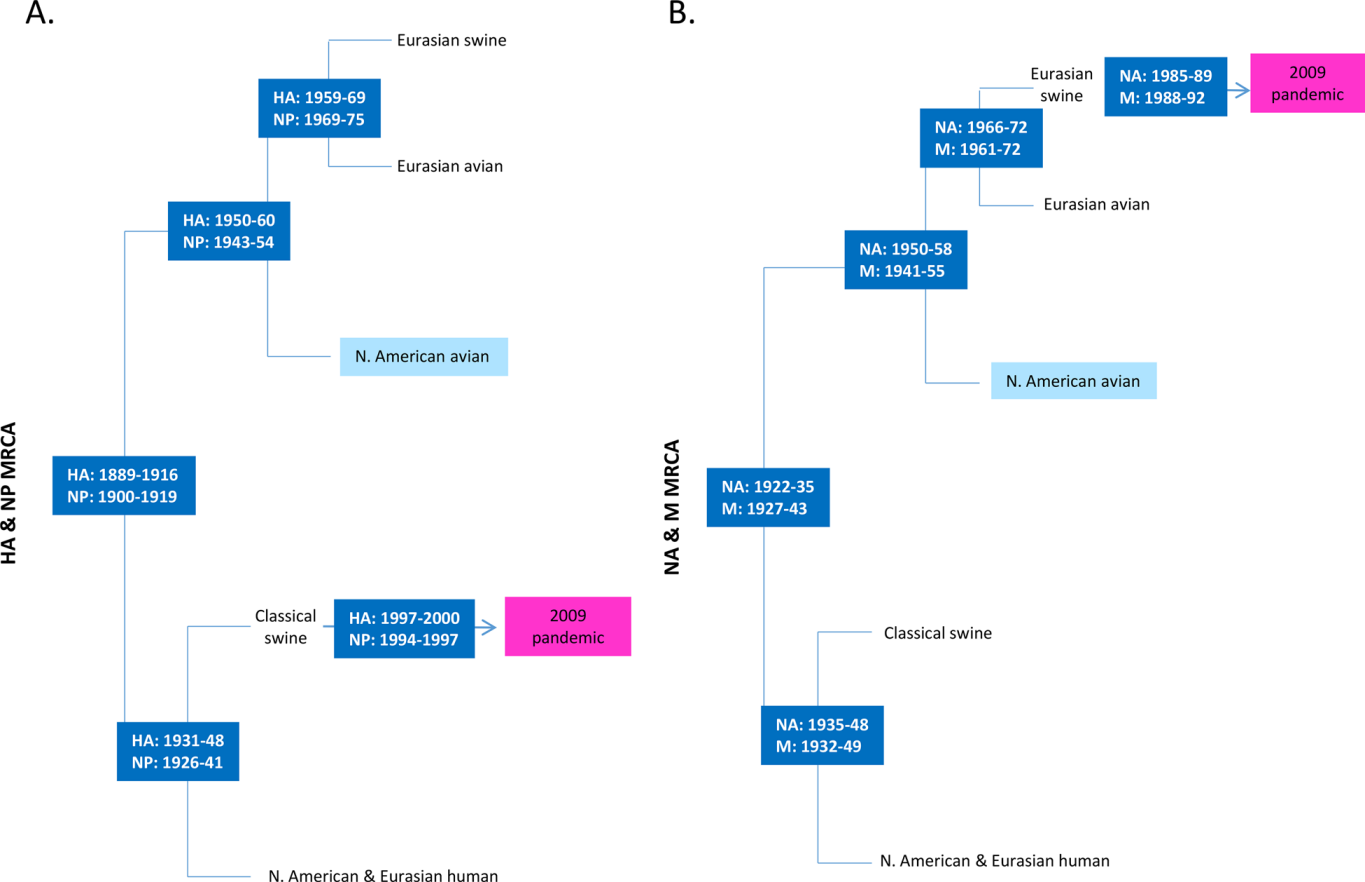
Schematic of the evolutionary history among HA, NP, NA and M segments of H1N1 IAVs. The schematic view is based on the phylogenetic trees of avian, swine, and human origin H1N1 IAVs and the most recent common ancestor (MRCA) for (A) HA and NP gene segments and (B) NA and M gene segments. North American avian (light blue) and 2009 pandemic H1N1 viruses (pink) are highlighted.

The ancestral HA and NP gene segments of H1N1 IAVs existed around 1889–1916 and 1900–1919, respectively (Fig 4A and S2–S3 Figs). Unlike the polymerase genes, these segments form 2 distinct clades that are largely characterized by host type. One lineage led to the emergence of both North American and Eurasian avian and Eurasian avian—like swine viruses; the other lineage led to the emergence of North American and Eurasian human, classical swine, and the 2009 pandemic H1N1 IAVs (Fig 4A and S2–S3 Figs). The North American avian H1N1 IAVs diverged from the MRCA with Eurasian avian and avian—like swine viruses around the early 1950s and 1940s, respectively. The HA and NP gene segments of North American avian IAVs evolved separately from the mammalian H1N1 IAVs, including the 2009 pandemic H1N1 viruses. Although the clades are largely characterized by host type instead of geographic origin, as they were in the polymerase genes, within-host intercontinental gene exchange has frequently occurred in mammals. Also concordant with the evolution of the polymerase gene segments was the lack of evidence of intercontinental gene exchange in birds.

The evolutionary histories of the NA and M segments paralleled those of the HA and NP segments, with the exception of the H1N1 2009 pandemic strains (Fig 4B and S4–S5 Figs). The MRCA of the NA and M segments existed around 1922–1935 and 1927–1943, respectively. The basal-most split largely segregated isolates by host type. One lineage led to the emergence of human and classical swine descendants; the other led to the emergence of North American and Eurasian avian and Eurasian avian—like swine H1N1 IAVs that eventually seeded the 2009 pandemic viruses (Fig 4B and S4–S5 Figs). Within each of these clades, the NA an M gene segments of North American avian H1N1 IAVs diverged from the MRCA with Eurasian avian and avian—like swine viruses around the mid-1950s. Again, mammalian intrahost, intercontinental exchange of NA and M gene segments has occurred frequently, whereas the only case of avian interhost gene exchange was from Eurasian avian to swine, which seeded the 2009 pandemic viruses (Fig 4B and S4–S5 Figs).

Overall, our phylogenetic analysis demonstrated that the evolution of the HA, NA, NP, and M gene segments has largely been host driven, with ready intercontinental exchange between mammalian hosts (Fig 4). The primary difference between the evolutionary histories of these pairs of segments is the topologic similarity of HA and NP on one hand, and NA and M on the other. The topologic differences between these pairs were due to the source of the 2009 pandemic strain, which was classical swine—like in the HA-NP gene segment pair, and Eurasian avian—like swine H1N1 in the NA-M gene segment pair. The similarity between the evolutionary histories of the gene segments within each pair suggests coevolution of each internal gene segment with its glycoprotein partner.

### Two alleles of the NS gene segment also circulate in H1N1 IAVs

The circulation of two alleles of NS gene segments (Allele A and Allele B) was previously described [25,26]. The NS gene segments of H1N1 IAVs diverged from the MRCA between 1603 and 1751 into two distinct lineages: one lineage resulted in NS gene segments circulating only in the avian population; the other led to the NS gene segment’s descendants circulating in avian, swine, human, and eventually the 2009 pandemic viruses. The NS gene segments of avian, swine, and human H1N1 IAVs in the latter lineage shared a MRCA until the early 1940s (S6 Fig). Eurasian avian and avian—like swine H1N1 IAVs diverged from North American avian H1N1 IAVs in the late 1940s. Since then, the NS segment of North American avian H1N1 IAVs belonging to allele B [25] has been evolving distinctly from all other NS gene segments circulating in human, swine, or Eurasian avian H1N1 IAVs.

### H1N1 IAVs of Charadriiformes origin might lead to the emergence of a new gene pool

Based on the surveillance data from Delaware Bay, no H1N1 subtype was detected in Charadriiformes species from 1976 to 2009 [27]. The appearance of H1N1 in Charadriiformes during a pandemic year led us to investigate the genetic relatedness of these viruses to the avian H1N1 IAV of Anseriformes origin and to the mammalian H1N1 IAVs, including the 2009 pandemic virus.

The 6 avian H1N1 IAVs of Charadriiformes origin are closely related to each other in their HA and NA gene segments. However, they show variations in their internal gene segments (PB2, PB1, PA, NP, M, NS). For instance, 5 avian H1N1 IAVs of Charadriiformes origin (A/shorebird/DE/170/2009, A/shorebird/DE/300/2009, A/shorebird/DE/318/2009, A/shorebird/DE/324/2009, and A/gull/DE/428/2009) are closely related to each other in their internal gene segments, and A/shorebird/DE/274/2009 is distantly related. In fact, A/shorebird/DE/274/2009 is a reassortant virus that acquired HA and NA gene segments from the H1N1 IAVs of Charadriiformes origin, whereas internal gene segments originated from H1N1 IAVs of Anseriformes origin (Figs 1 and 3 and S1–S6 Figs). The NS gene segments of all 6 H1N1 IAVs of Charadriiformes origin belong to Allele B; however, they all fall into separate clades nested within a North American avian clade (S6 Fig).

### Similarities among the amino acid residues at the HA antigenic sites of 31 North American avian H1N1 IAVs and those of seasonal and pandemic human H1N1 IAVs

To identify the HA antigenic structure of the 2009 pandemic H1N1 virus, the antigenic sites of the 1918 and 2009 pandemic H1N1 IAVs were previously compared with those of human H1N1 IAVs [28]. On the basis of the data published in Igarashi et al. (2010) [28], we compared the amino acid residues at the antigenic sites on the HA of 31 H1N1 IAVs of Charadriiformes and Anseriformes origins at St. Jude influenza repository (S2 Table). Of the 50 residues at 4 antigenic sites (Ca, Cb, Sa, and Sb) in the HA, 14 were fully conserved among human and avian H1N1 IAVs, and 9 were largely conserved (i.e., only a few human H1N1 strains had an amino acid substitution but reverted back to the residue in 1918 virus) (Table 1 and S2 Table). The remaining 27 residues showed variation among the pandemic, human, and avian H1N1 viruses over the years. Among those, 12 were conserved in avian (Anseriformes and Charadriiformes) and pandemic viruses (1918 and 2009) but not in human seasonal H1N1 IAVs (Table 1). Two antigenic sites (Sa and Ca) had an amino acid substitution (A212I in Sa and A158V in Ca) in the H1N1 IAVs of Charadriiformes origin, though both were conserved among pandemic and avian H1N1 IAVs of Anseriformes origin. However, H1N1 IAVs of Charadriiformes origin were identical to the 2009 pandemic virus at 2 other residues, N173 in Sa and P154 in Ca (S2 Table). Overall, approximately 60% to 80% of the amino acids in the 4 antigenic sites in the HA of 31 avian H1N1 IAVs were identical to those in the 1918 and/or 2009 pandemic virus; only 46% of the amino acid residues in the HA of human seasonal H1N1 IAVs were identical to those in the pandemic viruses.

**Table 1:** The antigenic sites and attributes of amino acids in the HA of avian H1N1 IAVs.

Attributes of amino acids	Number of amino acids at antigenic sites				
	Sa	Sb	Ca	Cb	Total
fully conserved	4	2	6	2	14
largely conserved*	5	0	4	0	9
1918 & 2009 avian-like (not human-like)	3	6^‡^	1^‡^	2	12
1918-like only	0	0	2	1	3
2009-like only	1	0	0	0	1
avian-like^†^	0	3	5	1	9
Total	13	11	18	6	48
Revised total^‡^	13	12	19	6	50

*Residues were considered largely conserved when the same amino acid residue observed in many isolates over the years.

^†^“avian-like” category indicates the number of amino acids at the HA antigenic sites of 31 avian IAVs of Anseriformes and Charadriiformes origin that were not seen in seasonal or pandemic human H1N1 IAVs.

^‡^Positions 158 in Ca and 212 in Sb are “pandemic-like only” in the H1N1 IAVs of Anseriformes origin but not in those of Charadriiformes origin. The total number of amino acids is 50 with the addition of these 2 positions.

## Discussion

H1N1 is the main subtype of IAVs cocirculating in avian, swine and human populations. It has been proposed that the 1918 pandemic was caused by a purely avian H1N1 virus [16]. The 2009 pandemic H1N1 virus had a unique gene constellation of avian, swine, and human origin, though all gene segments originated from birds prior to establishment in swine [14]. Thus, it is important to address the evolutionary dynamics within the H1N1 subtype of avian, swine, and human viruses to track the contribution of the avian H1N1 IAV gene pool to swine and human H1N1 IAVs in an effort to prepare for possible pandemics in the future. Additionally, it is important to understand the sudden emergence of H1N1 IAVs in the birds of Charadriiformes origin in 2009 to assess their role as a transitory host in the process of pandemic virus formation. On the basis of our previous study of the pathogenicity of 31 North American avian H1N1 IAVs in mice [17], we also manually inspected whether the avian H1N1 IAVs of similar pathogenicity are closely related.

Among the 8 influenza gene segments, PB2 and PA appeared to follow a more complicated evolutionary process than the other 6 segments; the introduction from avian to mammalian H1N1 IAVs has continued over the years. The contribution of avian PB2 and PA gene segments to the formation of 2009 pandemic was also previously shown [15]. We detected two main gene pools for PB2 and PA circulating in North American avian H1N1 IAVs: one is only in the avian population, and the other is more closely related to North American classical swine and the 2009 pandemic H1N1 IAVs. There were not as many introductions of PB1 as there were of PB2 and PA from the avian to swine population, yet avian PB1 introduced to classical swine eventually seeded the PB1 of the 2009 H1N1 IAVs. Therefore, the polymerase gene segments circulating in avian H1N1 IAVs have contributed to the gene pool circulating in the swine population that ultimately became the polymerase gene segments of the 2009 pandemic H1N1 IAVs.

Reconciling the tree topologies with host and geographic origin of the isolates, we concluded that there has been intercontinental exchange among the classical swine and human H1N1 polymerase gene segments, whereas avian H1N1 IAVs have evolved separately based on geography. This could be due to humans travelling across continents and the virus being readily transmitted between swine and human populations. In contrast, the bird flyways of America are geographically more isolated than those in Asia and Europe.

It has been speculated that the gene segments of the 2009 pandemic virus circulated for a long time, without being detected, before the virus emerged as a pandemic [14,15]. Those gene segments were contributed by multiple sources: the polymerase genes were from triple-reassortant swine viruses; the HA, NP, and NS were from classical swine viruses; and the NA and M were from Eurasian avian—like swine viruses [14,15]. Our study demonstrated that the topologies of the HA-NP and NA-M gene segment pairs were quite similar across all H1N1 IAVs. Moreover, those gene segments appear to have coevolved within the H1N1 subtype, including the gene segments of the 2009 pandemic virus. For instance, the 2009 pandemic virus acquired its HA and NP segments from classical swine viruses and its NA and M segments from Eurasian avian—like swine viruses. This is consistent with the earlier findings on the origin of 2009 pandemic virus gene segments [14,15]. Therefore, the coevolution and concurrent movement of some gene pairs between various hosts might be an important step in the emergence of past and future H1N1 pandemics. On the basis of this finding, we speculate that the coevolution of the NA and M gene segments has functional significance in the life cycle of the influenza virus. M1 protein is involved in virus assembly and budding, and NA is involved in the release of the virus from the host cell’s membrane.

H13 and H16 are unique subtypes of IAVs that circulate in Charadriiformes species [10]. The HA and NA gene segments of the 6 avian H1N1 IAVs of Charadriiformes origin from the St. Jude influenza repository were almost identical, with only slight differences. However, the viruses showed multiple genotypes and variations in their internal gene segments. For instance, A/shorebird/DE/274/2009 is a reassortant virus with an HA and NA from H1N1 IAVs of Charadriiformes origin and internal gene segments from H1N1 IAVs of Anseriformes origin. A/gull/DE/428/2009 also showed more variation than the other viruses, including in its HA gene segment. This finding indicates the importance of the host in virus evolution, not only at the order level (e.g. Charadriiformes versus Anseriformes) but also at the family level (Scolopacidae versus Laridae). Although the appearance of H1N1 IAVs in Charadriiformes was not recorded until 2009 [27], these 6 avian H1N1 viruses did not share a MRCA with the 2009 pandemic H1N1 isolates. However, the amino acid sequences of their HA antigenic sites were 60% to 80% identical to those of the 1918 and 2009 pandemic viruses, as were the H1N1 IAVs of Anseriformes origin in our study. We did not detect any significant difference between the viruses of Charadriiformes and Anseriformes origins based on the antigenic sites. The viruses of Charadriiformes origin were identical to the 2009 pandemic viruses at two additional residues at two antigenic sites (P154 in Ca and N173 in Sa); however, almost half of the H1N1 IAVs of Anseriformes origin in our study also carried an N173 in the Sa antigenic site.

On the basis of the pathogenicity of 31 North American avian H1N1 IAVs in a mouse model, we could not observe a relation between the phylogeny and the pathogenicity phenotype of the viruses. Although this may reflect the small number of viruses with known pathogenicity in our dataset, it may also indicate that phylogeny is independent of pathogenicity. If pathogenicity is caused by disparate mutations that have a combinatorial effect, such an effect would be difficult to detect by phylogenetic analysis. Thus, phylogenetic analysis may not be the best approach to predicting the virulence of influenza viruses that may be resulted from the genetic changes in multiple gene segments, reassortment, and/or the host origin of the virus.

Because the main concern for public health has (traditionally) been the highly pathogenic H5N1 viruses and other subtypes of emerging influenza viruses, mainly those in Asia and the Middle East (e.g., H7N9 and H9N2), our knowledge of the H1N1 IAVs circulating in wild birds is insufficient. However, this study emphasizes the significance of avian H1N1 IAVs in the evolutionary history of swine and pandemic viruses. Based on surveillance studies done at St. Jude, only approximately 6% of IAVs isolated from wild birds are of the H1N1 subtype. Although not a common subtype in birds, avian H1N1 IAV is important, in terms of its contributions to the gene pool circulating in mammalian IAVs. Our phylogenetic analysis indicated that there has been continuous introduction of avian polymerase gene segments into swine influenza viruses, and such reassortments can potentially lead to the emergence of an H1N1 pandemic. Thus, the H1N1 IAVs circulating in wild birds continue to pose a threat to swine and human populations. Charadriiformes birds can also serve as a transitory host for the emergence of new pandemics; an H1N1 AIV of Charadriiformes origin has been transmitted via respiratory droplets in the ferret model [17]. Consequently, close attention should be paid to all H1N1 virus isolates obtained during future surveillance studies for risk assessment and pandemic preparedness.

## Supporting Information

S1 Fig Evolutionary relatedness of PB1 gene segments of H1N1 IAVs of avian, swine and human origins isolated from North America and Eurasia. The taxa are colored based on their host and geography: red-North American avian isolates from the St. Jude repository, light blue-North American avian isolates, purple-Eurasian avian isolates, green- swine isolates from North America, grey- swine isolates from Eurasia (both Eurasian avian-like swine and Eurasian classical swine), dark blue-human isolates from North America; orange- human isolates from Eurasia, and pink- 2009 pandemic isolates.
(TIF)

S2 Fig Evolutionary relatedness of HA gene segments of H1N1 IAVs of avian, swine and human origins isolated from North America and Eurasia. The taxa are colored based on their host and geography: red-North American avian isolates from the St. Jude repository, light blue-North American avian isolates, purple-Eurasian avian isolates, green- swine isolates from North America, grey- swine isolates from Eurasia (both Eurasian avian-like swine and Eurasian classical swine), dark blue-human isolates from North America; orange- human isolates from Eurasia, and pink- 2009 pandemic isolates.
(TIF)

S3 Fig Evolutionary relatedness of NP gene segments of H1N1 IAVs of avian, swine and human origins isolated from North America and Eurasia. The taxa are colored based on their host and geography: red-North American avian isolates from the St. Jude repository, light blue-North American avian isolates, purple-Eurasian avian isolates, green- swine isolates from North America, grey- swine isolates from Eurasia (both Eurasian avian-like swine and Eurasian classical swine), dark blue-human isolates from North America; orange- human isolates from Eurasia, and pink- 2009 pandemic isolates.
(TIF)

S4 Fig Evolutionary relatedness of NA gene segments of H1N1 IAVs of avian, swine and human origins isolated from North America and Eurasia. The taxa are colored based on their host and geography: red-North American avian isolates from the St. Jude repository, light blue-North American avian isolates, purple-Eurasian avian isolates, green- swine isolates from North America, grey- swine isolates from Eurasia (both Eurasian avian-like swine and Eurasian classical swine), dark blue-human isolates from North America; orange- human isolates from Eurasia, and pink- 2009 pandemic isolates.
(TIF)

S5 Fig Evolutionary relatedness of M gene segments of H1N1 IAVs of avian, swine and human origins isolated from North America and Eurasia. The taxa are colored based on their host and geography: red-North American avian isolates from the St. Jude repository, light blue-North American avian isolates, purple-Eurasian avian isolates, green- swine isolates from North America, grey- swine isolates from Eurasia (both Eurasian avian-like swine and Eurasian classical swine), dark blue-human isolates from North America; orange- human isolates from Eurasia, and pink- 2009 pandemic isolates.
(TIF)

S6 Fig Evolutionary relatedness of NS gene segments of H1N1 IAVs of avian, swine and human origins isolated from North America and Eurasia. The taxa are colored based on their host and geography: red-North American avian isolates from the St. Jude repository, light blue-North American avian isolates, purple-Eurasian avian isolates, green- swine isolates from North America, grey- swine isolates from Eurasia (both Eurasian avian-like swine and Eurasian classical swine), dark blue-human isolates from North America; orange- human isolates from Eurasia, and pink- 2009 pandemic isolates.
(TIF)

S1 Table H1N1 IAVs of avian, swine, and human origin from North America and Eurasia that were used in this study and their GenBank accession numbers for 8 gene segments. (PDF)

S2 Table Amino acid substitutions at the HA antigenic sites in avian, human, and pandemic H1N1 IAVs. (PDF)
