# Supplementary figures and images for: The Genomic Contributions of Avian H1N1 Influenza A Viruses to the Evolution of Mammalian Strains
Source: PLoS One. 2015 Jul 24;10(7):e0133795. doi: 10.1371/journal.pone.0133795 (PMC4514870; doi:10.1371/journal.pone.0133795)

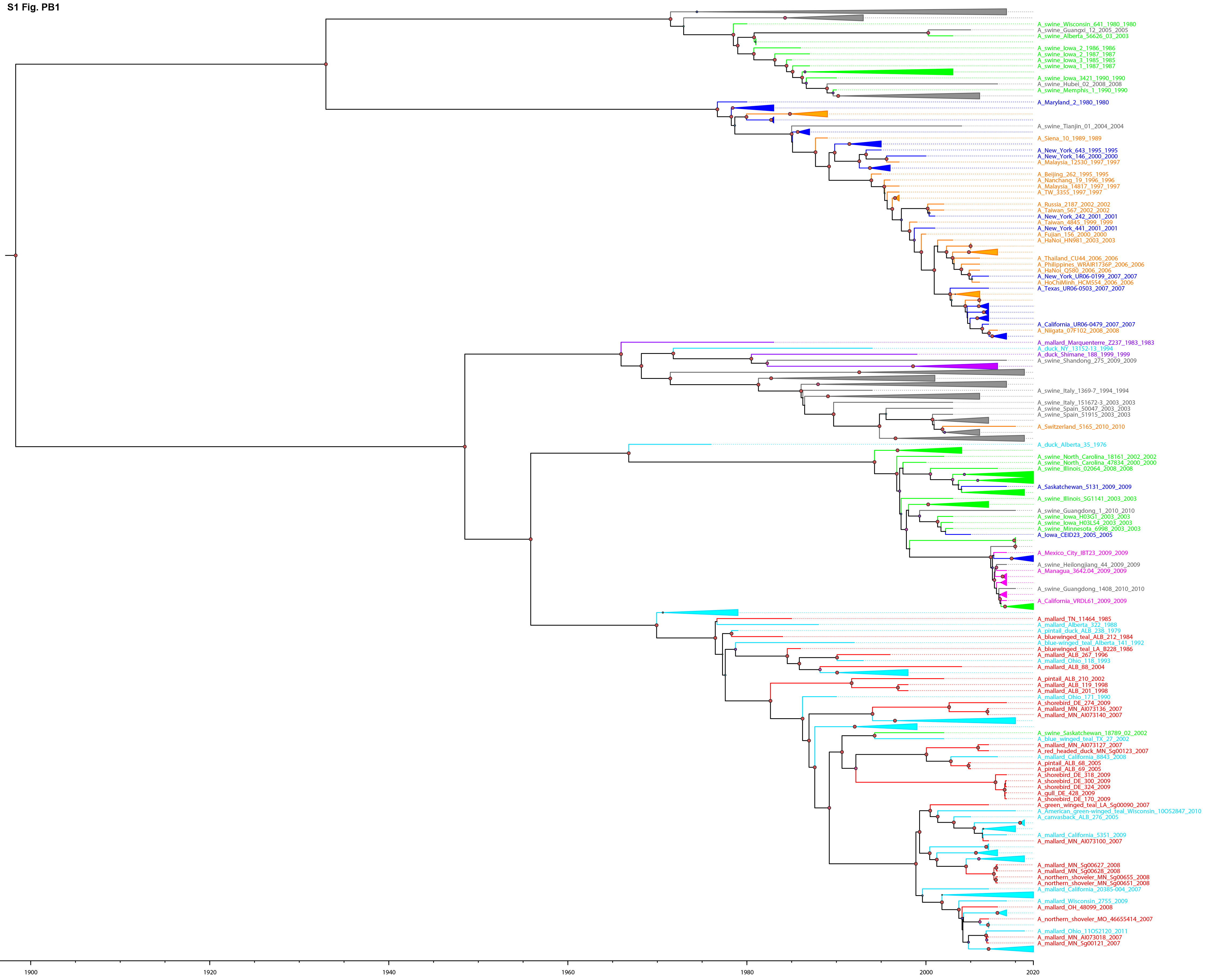

Supplement: S1 Fig — The taxa are colored based on their host and geography: red-North American avian isolates from the St. Jude repository, light blue-North American avian isolates, purple-Eurasian avian isolates, green- swine isolates from North America, grey- swine isolates from Eurasia (both Eurasian avian-like swine and Eurasian classical swine), dark blue-human isolates from North America; orange- human isolates from Eurasia, and pink- 2009 pandemic isolates. (TIF) [file pone.0133795.s001.tif]

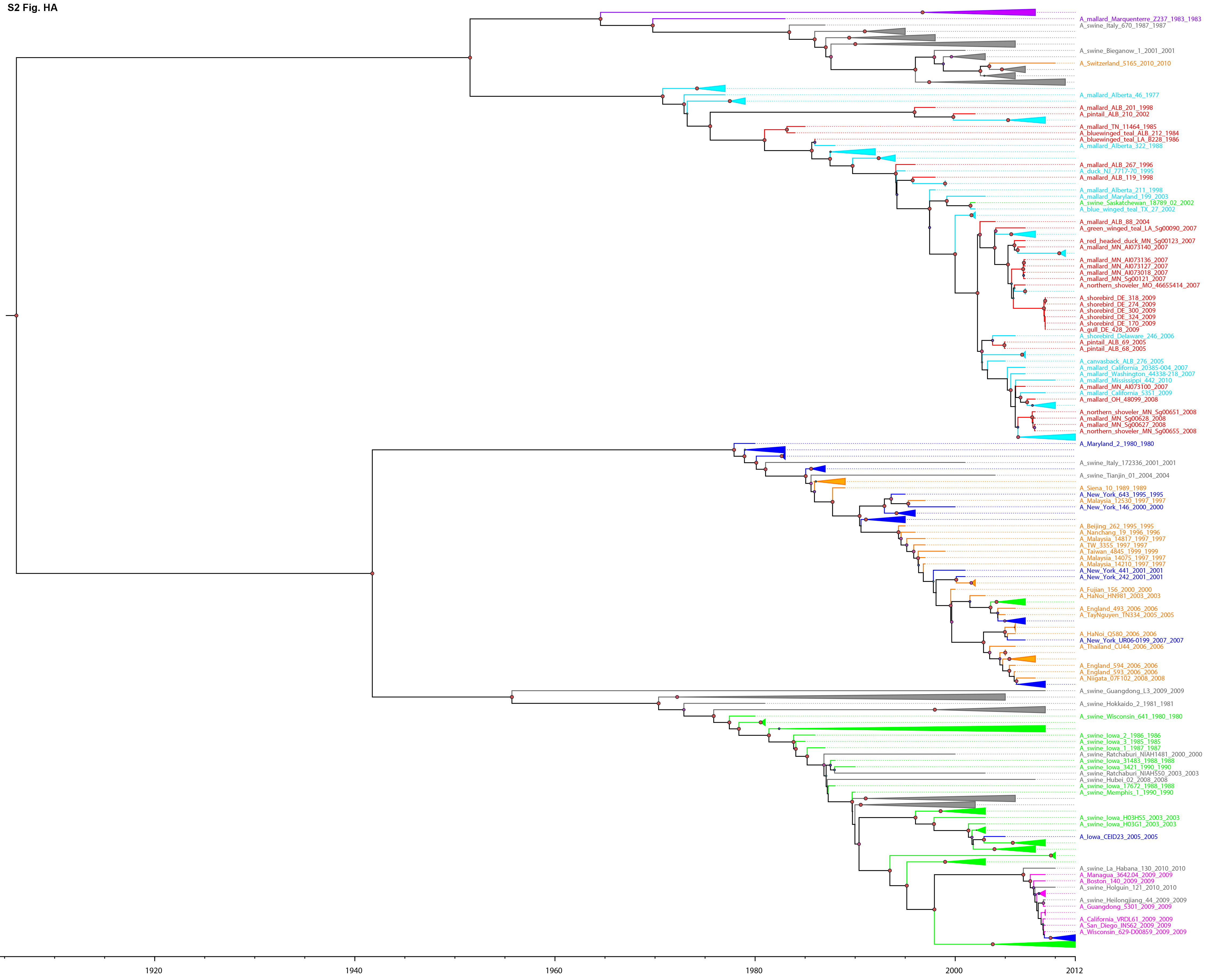

Supplement: S2 Fig — The taxa are colored based on their host and geography: red-North American avian isolates from the St. Jude repository, light blue-North American avian isolates, purple-Eurasian avian isolates, green- swine isolates from North America, grey- swine isolates from Eurasia (both Eurasian avian-like swine and Eurasian classical swine), dark blue-human isolates from North America; orange- human isolates from Eurasia, and pink- 2009 pandemic isolates. (TIF) [file pone.0133795.s002.tif]

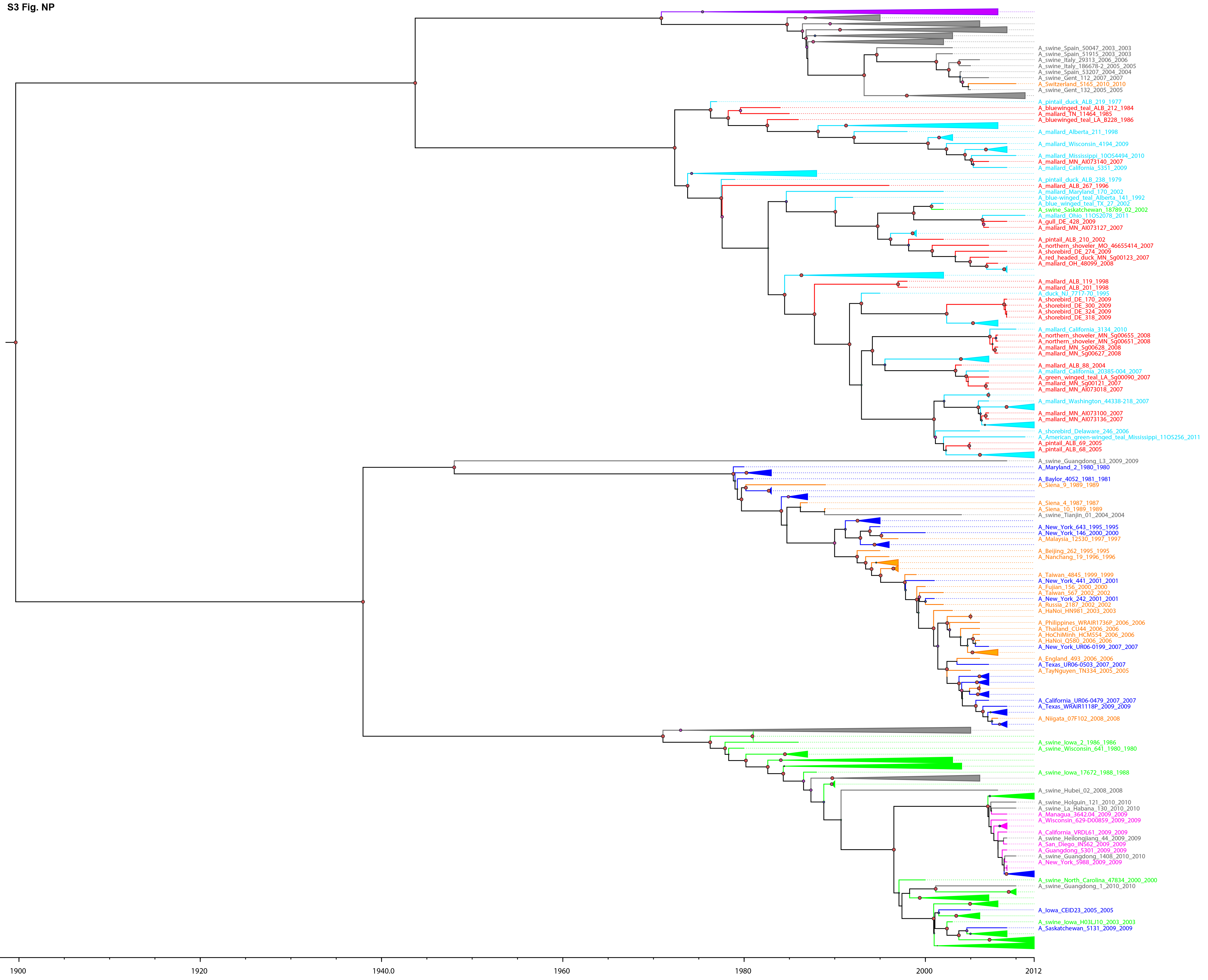

Supplement: S3 Fig — The taxa are colored based on their host and geography: red-North American avian isolates from the St. Jude repository, light blue-North American avian isolates, purple-Eurasian avian isolates, green- swine isolates from North America, grey- swine isolates from Eurasia (both Eurasian avian-like swine and Eurasian classical swine), dark blue-human isolates from North America; orange- human isolates from Eurasia, and pink- 2009 pandemic isolates. (TIF) [file pone.0133795.s003.tif]

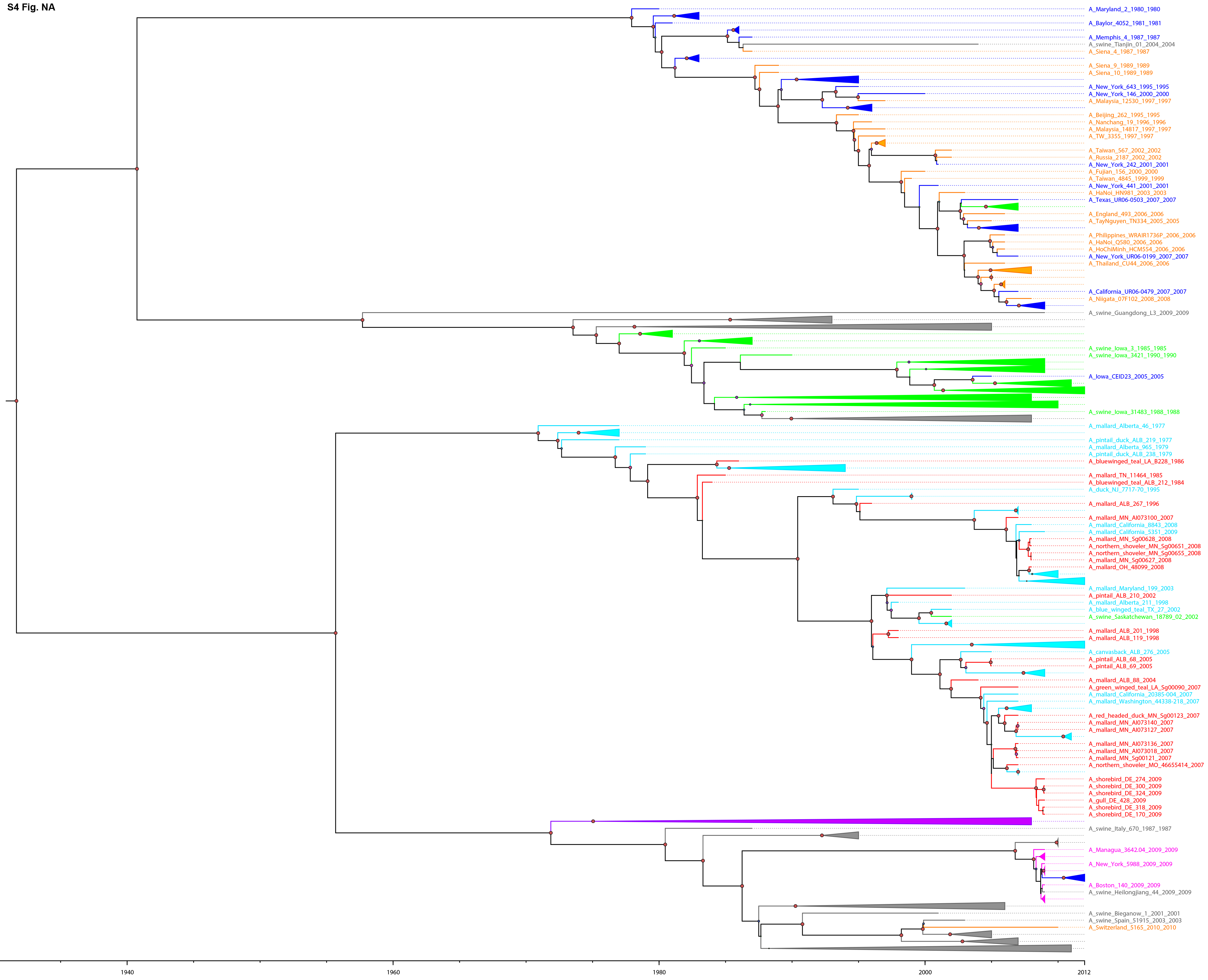

Supplement: S4 Fig — The taxa are colored based on their host and geography: red-North American avian isolates from the St. Jude repository, light blue-North American avian isolates, purple-Eurasian avian isolates, green- swine isolates from North America, grey- swine isolates from Eurasia (both Eurasian avian-like swine and Eurasian classical swine), dark blue-human isolates from North America; orange- human isolates from Eurasia, and pink- 2009 pandemic isolates. (TIF) [file pone.0133795.s004.tif]

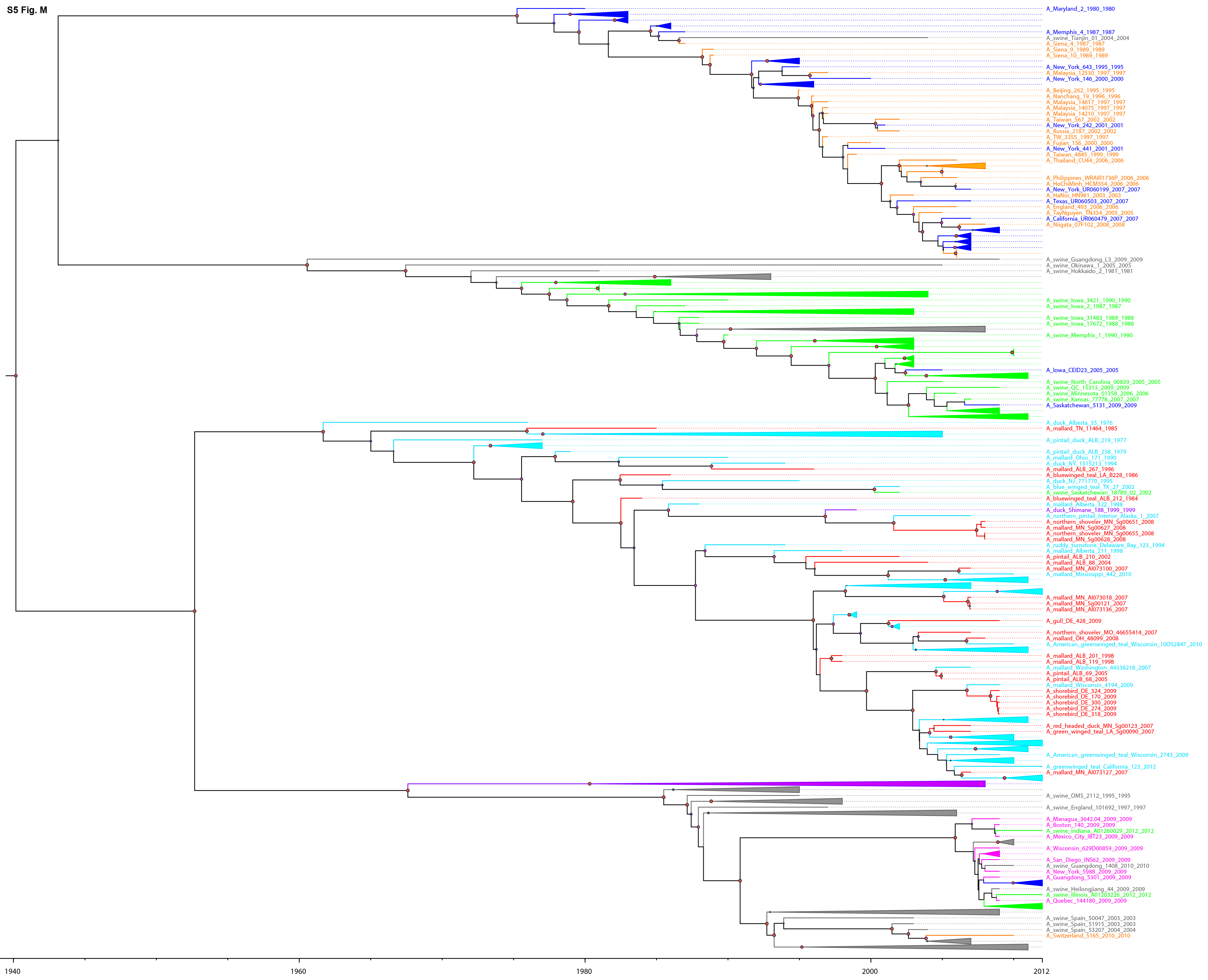

Supplement: S5 Fig — The taxa are colored based on their host and geography: red-North American avian isolates from the St. Jude repository, light blue-North American avian isolates, purple-Eurasian avian isolates, green- swine isolates from North America, grey- swine isolates from Eurasia (both Eurasian avian-like swine and Eurasian classical swine), dark blue-human isolates from North America; orange- human isolates from Eurasia, and pink- 2009 pandemic isolates. (TIF) [file pone.0133795.s005.tif]

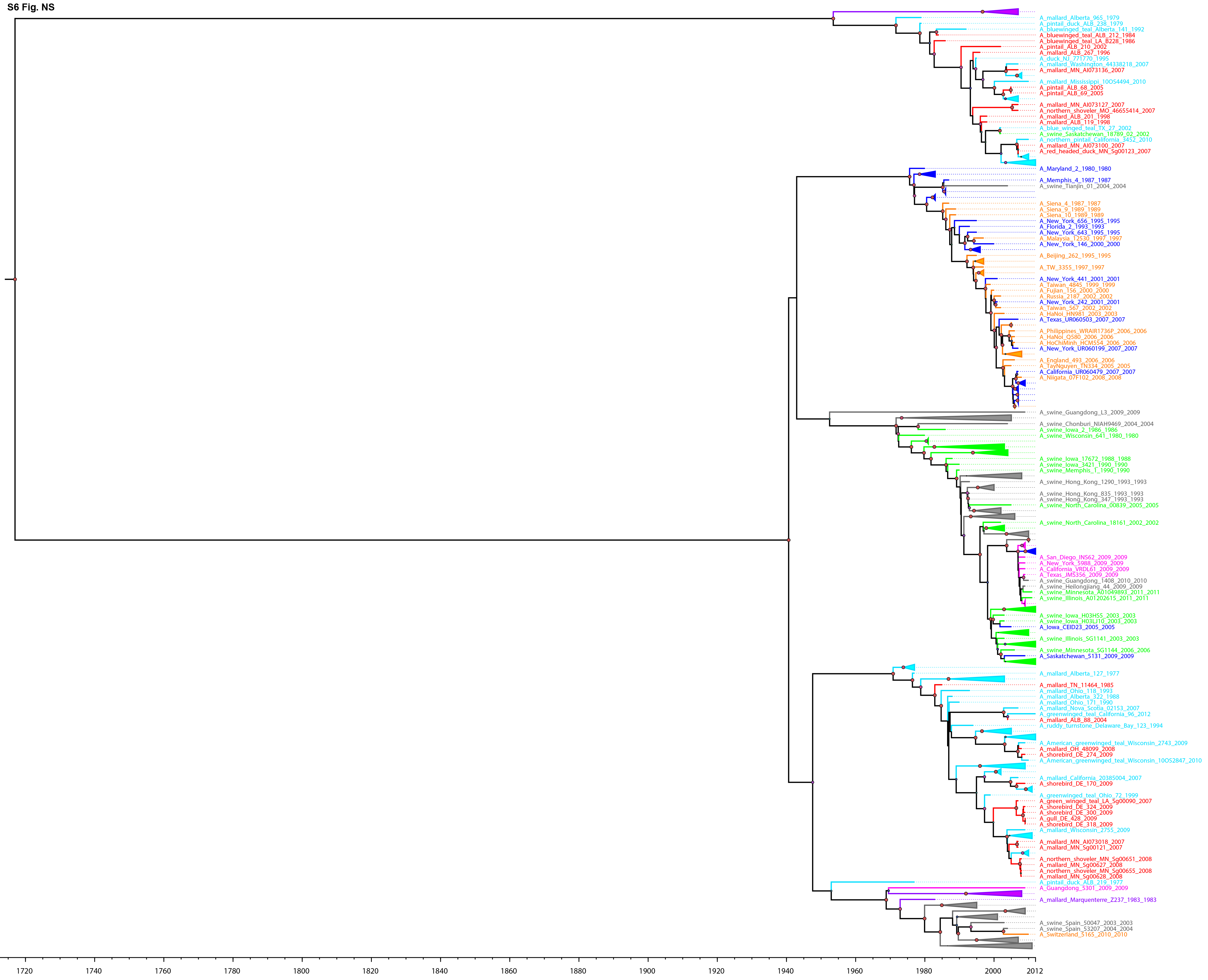

Supplement: S6 Fig — The taxa are colored based on their host and geography: red-North American avian isolates from the St. Jude repository, light blue-North American avian isolates, purple-Eurasian avian isolates, green- swine isolates from North America, grey- swine isolates from Eurasia (both Eurasian avian-like swine and Eurasian classical swine), dark blue-human isolates from North America; orange- human isolates from Eurasia, and pink- 2009 pandemic isolates. (TIF) [file pone.0133795.s006.tif]
