# Supplementary material for: The Genomic Contributions of Avian H1N1 Influenza A Viruses to the Evolution of Mammalian Strains
Source: PLoS One. 2015 Jul 24;10(7):e0133795. doi: 10.1371/journal.pone.0133795 (PMC4514870; doi:10.1371/journal.pone.0133795)
Supplement: S1 Table — (PDF) [file pone.0133795.s007.pdf]

S1 Table. H1N1 IAVs of avian, swine, and human origin from North America and Eurasia that were used in this study and their GenBank accession numbers for 8 gene segments.

geography/host origin[a]	taxon	PB2	PB1	PA	HA	NP	NA	M	NS	pathogenicity index[b]
North American avian/St. Jude	A/mallard/ALB/119/1998	KF424175	KF424176	KF424177	KF424178	KF424179	KF424180	KF424181	KF424182	4
	A/mallard/MN/AI07-3100/2007	KF424015	KF424016	KF424017	KF424018	KF424019	KF424020	KF424021	KF424022	4
	A/shorebird/DE/300/2009	KF424127	KF424128	KF424129	KF424130	KF424131	KF424132	KF424133	KF424134	4
	A/mallard/ALB/201/1998	KF424047	KF424048	KF424049	KF424050	KF424051	KF424052	KF424053	KF424054	4
	A/pintail/ALB/210/2002	KF424111	KF424112	KF424113	KF424114	KF424115	KF424116	KF424117	KF424118	4
	A/mallard/OH/4809-9/2008	KF424151	KF424152	KF424153	KF424154	KF424155	KF424156	KF424157	KF424158	4
	A/mallard/MN/AI07-3136/2007	KF424167	KF424168	KF424169	KF424170	KF424171	KF424172	KF424173	KF424174	4
	A/mallard/MN/AI07-3127/2007	KF424207	KF424208	KF424209	KF424210	KF424211	KF424212	KF424213	KF424214	4
	A/shorebird/DE/324/2009	KF424079	KF424080	KF424081	KF424082	KF424083	KF424084	KF424085	KF424086	4
	A/mallard/ALB/88/2004	KF424039	KF424040	KF424041	KF424042	KF424043	KF424044	KF424045	KF424046	4
	A/red headed duck/MN/Sg-00123/2007	KF424191	KF424192	KF424193	KF424194	KF424195	KF424196	KF424197	KF424198	3
	A/pintail/ALB/68/2005	KF424215	KF424216	KF424217	KF424218	KF424219	KF424220	KF424221	KF424222	3
	A/pintail/ALB/69/2005	KF424103	KF424104	KF424105	KF424106	KF424107	KF424108	KF424109	KF424110	3
	A/mallard/MN/Sg-00121/2007	KF424239	KF424240	KF424241	KF424242	KF424243	KF424244	KF424245	KF424246	3
	A/northern shoveler/MN/Sg-00651/2008	KF424031	KF424032	KF424033	KF424034	KF424035	KF424036	KF424037	KF424038	3
	A/gull/DE/428/2009	KF424023	KF424024	KF424025	KF424026	KF424027	KF424028	KF424029	KF424030	3
	A/northern shoveler/MN/Sg-00655/2008	KF424247	KF424248	KF424249	KF424250	KF424251	KF424252	KF424253	KF424254	3
	A/mallard/ALB/267/1996	KF424095	KF424096	KF424097	KF424098	KF424099	KF424100	KF424101	KF424102	3
	A/shorebird/DE/170/2009	KF424071	KF424072	KF424073	KF424074	KF424075	KF424076	KF424077	KF424078	3
	A/northern shoveler/MO/466554-14/2007	KF424119	KF424120	KF424121	KF424122	KF424123	KF424124	KF424125	KF424126	3
	A/mallard/MN/AI07-3018/2007	KF424199	KF424200	KF424201	KF424202	KF424203	KF424204	KF424205	KF424206	3
	A/blue winged teal/LA/B228/1986	KF424183	KF424184	KF424185	KF424186	KF424187	KF424188	KF424189	KF424190	3
	A/mallard/TN/11464/1985	KF424143	KF424144	KF424145	KF424146	KF424147	KF424148	KF424149	KF424150	3
	A/mallard/MN/AI07-3140/2007	KF424255	KF424256	KF424257	KF424258	KF424259	KF424260	KF424261	KF424262	3
	A/canvasback/ALB/276/2005	KF424159	KF424160	KF424161	KF424162	KF424163	KF424164	KF424165	KF424166	3
	A/blue winged teal/ALB/212/1984	KF424231	KF424232	KF424233	KF424234	KF424235	KF424236	KF424237	KF424238	3
	A/mallard/MN/Sg-00628/2008	KF424135	KF424136	KF424137	KF424138	KF424139	KF424140	KF424141	KF424142	3
	A/shorebird/DE/318/2009	KF424223	KF424224	KF424225	KF424226	KF424227	KF424228	KF424229	KF424230	2
	A/mallard/MN/Sg-00627/2008	KF424055	KF424056	KF424057	KF424058	KF424059	KF424060	KF424061	KF424062	2
	A/shorebird/DE/274/2009	KF424063	KF424064	KF424065	KF424066	KF424067	KF424068	KF424069	KF424070	2
	A/green winged teal/LA/Sg-00090/2007	KF424087	KF424088	KF424089	KF424090	KF424091	KF424092	KF424093	KF424094	1
North American avian	A/American black duck/New Brunswick/01989/2007	CY129381	CY129380	CY129379	CY129374	CY129377	CY129376	CY129375	CY129378	
	A/American green-winged teal/Mississippi/11OS256/2011	CY133692	CY133691	CY133690	CY133685	CY133688	CY133687	CY133686	CY133689	
	A/American green -winged teal/Wisconsin/10OS2847/2010	CY133092	CY133091	CY133090	CY133085	CY133088	CY133087	CY133086	CY133089	
	A/American green-winged teal/Wisconsin/2743/2009	CY097509	CY097508	CY097507	CY097502	CY097505	CY097504	CY097503	CY097506	
	A/blue-winged teal/Alberta/141/1992	CY004545	CY004544	CY004543	CY004539	CY004541	CY014568	CY004540	CY004542	
	A/blue winged teal/TX/27/2002	FJ357060	FJ357059	FJ357058	FJ357053	FJ357056	FJ357055	FJ357054	FJ357057	

A/duck/Alberta/35/1976	CY130013	CY130012	CY130011	CY130006	CY130009	CY130008	CY130007	CY130010
A/duck/Interior Alaska/7MP1582/2007	CY078994	CY078993	CY078992	CY078987	CY078990	CY078989	CY078988	CY078991
A/duck/NJ/7717-70/1995	EU026117	EU026116	EU026115	EU026110	EU026113	EU026112	EU026111	EU026114
A/duck/NY/13152-13/1994	EU026109	EU026108	EU026107	EU026102	EU026105	EU026104	EU026103	EU026106
A/green-winged teal/California/123/2012	CY134358	CY134357	CY134356	CY134351	CY134354	CY134353	CY134352	CY134355
A/green-winged/teal/California/2940/2011	CY134326	CY134325	CY134324	CY134319	CY134322	CY134321	CY134320	CY134323
A/green-winged/teal/California/96/2012	CY134350	CY134349	CY134348	CY134343	CY134346	CY134345	CY134344	CY134347
A/green-winged/teal/Ohio/72/1999	CY017724	CY017723	CY017722	CY017717	CY017720	CY017719	CY017718	CY017721
A/mallard/Alberta/127/1977	CY004599	CY004598	CY004597	CY004592	CY004595	CY004594	CY004593	CY004596
A/mallard/Alberta/211/1998	CY103740	CY103739	CY103738	CY103733	CY103736	CY103735	CY103734	CY103737
A/mallard/Alberta/322/1988	CY004538	CY004537	CY004536	CY004531	CY004534	CY004533	CY004532	CY004535
A/mallard/Alberta/46/1977	CY004473	CY004472	CY004471	CY004466	CY004469	CY004468	CY004467	CY004470
A/mallard/Alberta/965/1979	CY004497	CY004496	CY004495	CY004490	CY004493	CY004492	CY004491	CY004494
A/mallard/California/20385-004/2007	CY076140	CY076139	CY076138	CY076133	CY076136	CY076135	CY076134	CY076137
A/mallard/California/3134/2010	CY120618	CY120617	CY120616	CY120611	CY120614	CY120613	CY120612	CY120615
A/mallard/California/5351/2009	CY094708	CY094707	CY094706	CY094701	CY094704	CY094703	CY094702	CY094705
A/mallard/California/6768/2009	CY094556	CY094555	CY094554	CY094549	CY094552	CY094551	CY094550	CY094553
A/mallard/California/8843/2008	CY093662	CY093661	CY093660	CY093655	CY093658	CY093657	CY093656	CY093659
A/mallard/Interior Alaska/7MP0172/2007	CY078938	CY078937	CY078936	CY078931	CY078934	CY078933	CY078932	CY078935
A/mallard/Interior Alaska/7MP0747/2007	CY078962	CY078961	CY078960	CY078955	CY078958	CY078957	CY078956	CY078959
A/mallard/Interior Alaska/8BM2102/2008	CY080122	CY080121	CY080120	CY080115	CY080118	CY080117	CY080116	CY080119
A/mallard/Maryland/02-375/2002	EU743599	EU743598	EU743597	EU743592	EU743595	EU743594	EU743593	EU743596
A/mallard/Maryland/170/2002	EU026044	EU026043	EU026042	EU026037	EU026040	EU026039	EU026038	EU026041
A/mallard/Maryland/199/2003	EU980523	EU980522	EU980521	EU980516	EU980519	EU980518	EU980517	EU980520
A/mallard/Maryland/403/2002	EU026089	EU026088	EU026087	EU026082	EU026085	EU026084	EU026083	EU026086
A/mallard/Mississippi/10OS4494/2010	CY133305	CY133304	CY133303	CY133298	CY133301	CY133300	CY133299	CY133302
A/mallard/Mississippi/413/2010	CY097769	CY097768	CY097767	CY097762	CY097765	CY097764	CY097763	CY097766
A/mallard/Mississippi/442/2010	CY097777	CY097776	CY097775	CY097770	CY097773	CY097772	CY097771	CY097774
A/mallard/Nova Scotia/02153/2007	CY128925	CY128924	CY128923	CY128918	CY128921	CY128920	CY128919	CY128922
A/mallard/Ohio/118/1993	CY018892	CY018891	CY018890	CY018885	CY018888	CY018887	CY018886	CY018889
A/mallard/Ohio/11OS1991/2011	CY132252	CY132251	CY132250	CY132245	CY132248	CY132247	CY132246	CY132249
A/mallard/Ohio/11OS2001/2011	CY132532	CY132531	CY132530	CY132525	CY132528	CY132527	CY132526	CY132529
A/mallard/Ohio/11OS2078/2011	CY132236	CY132235	CY132234	CY132229	CY132232	CY132231	CY132230	CY132233
A/mallard/Ohio/11OS2120/2011	CY132564	CY132563	CY132562	CY132557	CY132560	CY132559	CY132558	CY132561
A/mallard/Ohio/11OS2232/2011	CY132244	CY132243	CY132242	CY132237	CY132240	CY132239	CY132238	CY132241
A/mallard/Ohio/171/1990	CY012807	CY012806	CY012805	CY012800	CY012803	CY012802	CY012801	CY012804
A/mallard/Ohio/66/1999	CY016962	CY016961	CY016960	CY016955	CY016958	CY016957	CY016956	CY016959
A/mallard/Washington/44338-218/2007	CY076644	CY076643	CY076642	CY076637	CY076640	CY076639	CY076638	CY076641
A/mallard/Wisconsin/2755/2009	CY097009	CY097008	CY097007	CY097002	CY097005	CY097004	CY097003	CY097006
A/mallard/Wisconsin/3165/2009	CY097389	CY097388	CY097387	CY097382	CY097385	CY097384	CY097383	CY097386
A/mallard/Wisconsin/4194/2009	CY097397	CY097396	CY097395	CY097390	CY097393	CY097392	CY097391	CY097394
A/mallard/Wisconsin/4197/2009	CY097413	CY097412	CY097411	CY097406	CY097409	CY097408	CY097407	CY097410
A/northern pintail/California/183/2012	CY134374	CY134373	CY134372	CY134367	CY134370	CY134369	CY134368	CY134371
A/northern pintail/California/3452/2010	CY120658	CY120657	CY120656	CY120651	CY120654	CY120653	CY120652	CY120655
A/northern pintail/Interior Alaska/1/2007	CY039744	CY039745	CY039746	CY039747	CY039748	CY039749	CY039750	CY039751
A/northern shoveler/California/138/2012	CY134366	CY134365	CY134364	CY134359	CY134362	CY134361	CY134360	CY134363
A/northern shoveler/Interior Alaska/7MP1077/2007	CY077301	CY077300	CY077299	CY077294	CY077297	CY077296	CY077295	CY077298
A/pintail/Alberta/21/2006	CY045398	CY045397	CY045396	CY045391	CY045394	CY045393	CY045392	CY045395
A/pintail duck/ALB/219/1977	CY004481	CY004480	CY004479	CY004474	CY004477	CY004476	CY004475	CY004478

	A/pintail duck/ALB/238/1979	CY004489	CY004488	CY004487	CY004482	CY004485	CY004484	CY004483	CY004486
	A/ruddy turnstone/Delaware Bay/123/1994	CY101941	CY101940	CY101939	CY101934	CY101937	CY101936	CY101935	CY101938
	A/shorebird/Delaware/246/2006	CY043911	CY043910	CY043909	CY043904	CY043907	CY043906	CY043905	CY043908
**North American swine**	A/swine/Alberta/56626/03/2003	DQ280197	DQ280198	DQ280199	DQ280203	DQ280201	DQ280202	DQ280204	DQ280200
	A/swine/Illinois/02064/2008	CY099102	CY099101	CY099100	CY099095	CY099098	CY099097	CY099096	CY099099
	A/swine/Illinois/02238/2008	CY099076	CY099075	CY099074	CY099069	CY099072	CY099071	CY099070	CY099073
	A/swine/Illinois/02251/2008	CY099262	CY099261	CY099260	CY099255	CY099258	CY099257	CY099256	CY099259
	A/swine/Illinois/02695/2009	CY099278	CY099277	CY099276	CY099271	CY099274	CY099273	CY099272	CY099275
	A/swine/Illinois/A00857129/2011	JX099362	JX099363	JX099364	JX099365	JX099366	JX099367	JX099368	JX099369
	A/swine/Illinois/A00857136c/2011	JX099386	JX099387	JX099388	JX099389	JX099390	JX099391	JX099392	JX099393
	A/swine/Illinois/A01049905/2011	JX045953	JX045954	JX045955	JN652506	JX045956	JN652540	JN652575	JX045957
	A/swine/Illinois/A01104011/2012	JX099394	JX099395	JX099396	JX099397	JX099398	JX099399	JX099400	JX099401
	A/swine/Illinois/A01202615/2011	KC508545	KC508546	KC508547	JX092309	KC508548	JX092358	JX092407	KC508549
	A/swine/Illinois/A01203226/2012	KC616632	KC616633	KC616634	JQ739699	KC616635	JQ739711	JQ739705	KC616636
	A/swine/Illinois/SG1141/2003	CY099198	CY099197	CY099196	CY099191	CY099194	CY099193	CY099192	CY099195
	A/swine/Indiana/A01202622/2011	KC508588	KC508589	KC508590	JX092312	KC508591	JX092361	JX092410	KC508592
	A/swine/Indiana/A01260029/2012	KC508565	KC508566	KC508567	KC167135	KC508568	KC167136	KC167137	KC508569
	A/swine/Iowa/1/1987	CY022969	CY022968	CY022967	CY022962	CY022965	CY022964	CY022963	CY022966
	A/swine/Iowa/17672/1988	CY022340	CY022339	CY022338	CY022333	CY022336	CY022335	CY022334	CY022337
	A/swine/Iowa/2/1986	CY096890	CY096889	CY096888	CY096883	CY096886	CY096885	CY096884	CY096887
	A/swine/Iowa/2/1987	CY028178	CY028177	CY028176	CY028171	CY028174	CY028173	CY028172	CY028175
	A/swine/Iowa/31483/1988	CY022977	CY022976	CY022975	CY022970	CY022973	CY022972	CY022971	CY022974
	A/swine/Iowa/3/1985	CY022332	CY022331	CY022330	CY022325	CY022328	CY022327	CY022326	CY022329
	A/swine/Iowa/3421/1990	CY096882	CY096881	CY096880	CY096875	CY096878	CY096877	CY096876	CY096879
	A/swine/Iowa/A01049210/2010	JQ738184	JQ738185	JQ738186	JQ398772	JQ738187	JQ398759	JQ398748	JQ738188
	A/swine/Iowa/A01049894/2011	JX045948	JX045949	JX045950	JN652505	JX045951	JN652538	JN652573	JX045952
	A/swine/Iowa/A01049950/2011	JX045958	JX045959	JX045960	JN652513	JX045961	JN652547	JN652581	JX045962
	A/swine/Iowa/A01267837/2012	KC616677	KC616678	KC616679	KC117391	KC616680	KC117393	KC117392	KC616681
	A/swine/Iowa/H03G1/2003	GU135907	GU135908	GU135909	GU135910	GU135911	GU135912	GU135913	GU135914
	A/swine/Iowa/H03HS5/2003	GU135930	GU135931	GU135932	GU135933	GU135934	GU135935	GU135936	GU135937
	A/swine/Iowa/H03LJ10/2003	GU135946	GU135947	GU135948	GU135949	GU135950	GU135951	GU135952	GU135953
	A/swine/Iowa/H03LS4/2003	GU135954	GU135955	GU135956	GU135957	GU135958	GU135959	GU135960	GU135961
	A/swine/Kansas/77778/2007	GQ484358	GQ484357	GQ484359	GQ484355	GQ484360	GQ484356	GQ484361	GQ484362
	A/swine/La Habana/130/2010	HE584753	HE584754	HE584755	HE584756	HE584757	HE584758	HE584759	HE584760
	A/swine/Memphis/1/1990	CY035077	CY035076	CY035075	CY035070	CY035073	CY035072	CY035071	CY035074
	A/swine/Mexico/Mex19/2010	CY122330	CY122331	CY122332	CY122333	CY122334	CY122335	CY122336	CY122337
	A/swine/Mexico/Ver29/2010	CY122386	CY122387	CY122388	CY122389	CY122390	CY122391	CY122392	CY122393
	A/swine/Michigan/A01202708/2011	KC508550	KC508551	KC508552	JX092421	KC508553	JX092461	JX092501	KC508554
	A/swine/Minnesota/001200/2006	CY099084	CY099083	CY099082	CY099077	CY099080	CY099079	CY099078	CY099081
	A/swine/Minnesota/01358/2006	CY099174	CY099173	CY099172	CY099167	CY099170	CY099169	CY099168	CY099171
	A/swine/Minnesota/02053/2008	CY099126	CY099125	CY099124	CY099119	CY099122	CY099121	CY099120	CY099123
	A/swine/Minnesota/02475/2008	CY099246	CY099245	CY099244	CY099239	CY099242	CY099241	CY099240	CY099243
	A/swine/Minnesota/02905/2009	CY099142	CY099141	CY099140	CY099135	CY099138	CY099137	CY099136	CY099139
	A/swine/Minnesota/07002083/2007	FJ611896	FJ611895	FJ611897	FJ611898	FJ611899	FJ611900	FJ611901	FJ611902
	A/swine/Minnesota/6998/2003	CY098481	CY098482	CY098483	CY098484	CY098485	CY098486	CY098487	CY098488
	A/swine/Minnesota/A01049893/2011	JX045943	JX045944	JX045945	JN652504	JX045946	JN652537	JN652572	JX045947
	A/swine/Minnesota/A01267908/2012	KC616682	KC616683	KC616684	KC167153	KC616685	KC167155	KC167154	KC616686
	A/swine/Minnesota/SG1144/2006	CY099206	CY099205	CY099204	CY099199	CY099202	CY099201	CY099200	CY099203
	A/swine/MN/23506/2009	HM125979	HM125980	HM125981	HM125982	HM125983	HM125984	HM125985	HM125986

	A/swine/North Carolina/00839/2005	CY099158	CY099157	CY099156	CY099151	CY099154	CY099153	CY099152	CY099155
	A/swine/North Carolina/18161/2002	CY098513	CY098514	CY098515	CY098516	CY098517	CY098518	CY098519	CY098520
	A/swine/North Carolina/3793/2008	JQ624664	JQ624665	JQ624666	JQ624667	JQ624668	JQ624669	JQ624670	JQ624671
	A/swine/North Carolina/47834/2000	CY098473	CY098474	CY098475	CY098476	CY098477	CY098478	CY098479	CY098480
	A/swine/North Dakota/A01104053/2012	KC435073	KC435074	KC435075	KC435076	KC435077	KC435078	KC435079	KC435080
	A/swine/OH/511445/2007	EU604691	EU604692	EU604693	EU604689	EU604694	EU604690	EU604695	EU604696
	A/swine/Ontario/11112/2004	DQ280245	DQ280246	DQ280247	DQ280250	DQ280249	DQ280251	DQ280252	DQ280248
	A/swine/Ontario/23866/2004	DQ280237	DQ280238	DQ280239	DQ280243	DQ280241	DQ280242	DQ280244	DQ280240
	A/swine/Ontario/3/1981	CY026450	CY026449	CY026448	CY026443	CY026446	CY026445	CY026444	CY026447
	A/swine/Ontario/4/1981	CY022388	CY022387	CY022386	CY022381	CY022384	CY022383	CY022382	CY022385
	A/swine/Ontario/53518/2003	DQ280213	DQ280214	DQ280215	DQ280219	DQ280217	DQ280218	DQ280220	DQ280216
	A/swine/Ontario/57561/2003	DQ280189	DQ280190	DQ280191	DQ280195	DQ280193	DQ280194	DQ280196	DQ280192
	A/swine/QC/1531-3/2009	JF713871	JF713872	JF713873	JF713874	JF713875	JF713876	JF713877	JF713878
	A/swine/Saskatchewan/18789/2002	AY619954	AY619955	AY619956	AY619961	AY619958	AY619960	AY619959	AY619957
	A/swine/Wisconsin/641/1980	CY022452	CY022451	CY022450	CY022445	CY022448	CY022447	CY022446	CY022449
	A/SW/MB/5-5/2009	JF714017	JF714018	JF714019	JF714020	JF714021	JF714022	JF714023	JF714024
**North American human**	A/Baylor/11515/1982	CY010371	CY010370	CY010369	CY010364	CY010367	CY010366	CY010365	CY010368
	A/Baylor/4052/1981	CY021036	CY021035	CY021034	CY021029	CY021032	CY021031	CY021030	CY021033
	A/California/UR06-0479/2007	CY028314	CY028313	CY028312	CY028307	CY028310	CY028309	CY028308	CY028311
	A/Florida/06/2012	JX905428	JX905427	JX905429	JX905426	JX905430	JX905423	JX905425	JX905424
	A/Florida/2/1993	CY125051	CY125050	CY125049	CY125044	CY125047	CY125046	CY125045	CY125048
	A/California/VRDL256/2009	CY074378	CY074377	CY074376	CY074371	CY074374	CY074373	CY074372	CY074375
	A/California/VRDL280/2009	CY074442	CY074441	CY074440	CY074435	CY074438	CY074437	CY074436	CY074439
	A/California/VRDL295/2009	CY074498	CY074497	CY074496	CY074491	CY074494	CY074493	CY074492	CY074495
	A/California/VRDL299/2009	CY074522	CY074521	CY074520	CY074515	CY074518	CY074517	CY074516	CY074519
	A/Illinois/UR06-0491/2007	CY026802	CY026801	CY026800	CY026795	CY026798	CY026797	CY026796	CY026799
	A/Iowa/CEID23/2005	DQ889682	DQ889683	DQ889684	DQ889689	DQ889686	DQ889687	DQ889688	DQ889685
	A/Kentucky/UR060-046/2007	CY028042	CY028041	CY028040	CY028035	CY028038	CY028037	CY028036	CY028039
	A/Kentucky/UR06-0539/2007	CY025946	CY025945	CY025944	CY025939	CY025942	CY025941	CY025940	CY025943
	A/Maryland/2/1980	CY020188	CY020187	CY020186	CY020181	CY020184	CY020183	CY020182	CY020185
	A/Memphis/11/1983	CY011311	CY011310	CY011309	CY011304	CY011307	CY011306	CY011305	CY011308
	A/Memphis/1/1983	CY012887	CY012886	CY012885	CY012880	CY012883	CY012882	CY012881	CY012884
	A/Memphis/12/1986	CY019108	CY019107	CY019106	CY019101	CY019104	CY019103	CY019102	CY019105
	A/Memphis/18/1983	CY010987	CY010986	CY010985	CY010980	CY010983	CY010982	CY010981	CY010984
	A/Memphis/2/1983	CY012895	CY012894	CY012893	CY012888	CY012891	CY012890	CY012889	CY012892
	A/Memphis/24/1983	CY017218	CY017217	CY017216	CY017211	CY017214	CY017213	CY017212	CY017215
	A/Memphis/3/1996	CY019124	CY019123	CY019122	CY019117	CY019120	CY019119	CY019118	CY019121
	A/Memphis/39/1983	CY020244	CY020243	CY020242	CY020237	CY020240	CY020239	CY020238	CY020241
	A/Memphis/4/1987	CY019786	CY019785	CY019784	CY019779	CY019782	CY019781	CY019780	CY019783
	A/Memphis/6/1983	CY015531	CY015530	CY015529	CY015524	CY015527	CY015526	CY015525	CY015528
	A/Memphis/8/1983	CY010947	CY010946	CY010945	CY010940	CY010943	CY010942	CY010941	CY010944
	A/New York/146/2000	CY000455	CY000456	CY000454	CY000449	CY000452	CY000451	CY000450	CY000453
	A/New York/242/2001	CY006362	CY006361	CY006360	CY006355	CY006358	CY006357	CY006356	CY006359
	A/New York/2924-1/1986	CY021740	CY021739	CY021738	CY021733	CY021736	CY021735	CY021734	CY021737
	A/New York/441/2001	CY009243	CY009242	CY009241	CY009236	CY009239	CY009238	CY009237	CY009240
	A/New York/614/1995	CY010811	CY010810	CY010809	CY010804	CY010807	CY010806	CY010805	CY010808
	A/New York/629/1995	CY011807	CY011806	CY011805	CY011800	CY011803	CY011802	CY011801	CY011804
	A/New York/633/1995	CY016970	CY016969	CY016968	CY016963	CY016966	CY016965	CY016964	CY016967
	A/New York/643/1995	CY013286	CY013285	CY013284	CY013279	CY013282	CY013281	CY013280	CY013283

	A/New York/656/1995	CY013310	CY013309	CY013308	CY013303	CY013306	CY013305	CY013304	CY013307
	A/New York/UR06-0199/2007	CY026634	CY026633	CY026632	CY026627	CY026630	CY026629	CY026628	CY026631
	A/Oregon/UR06-0185/2007	CY027962	CY027961	CY027960	CY027955	CY027958	CY027957	CY027956	CY027959
	A/Saskatchewan/5131/2009	GQ457544	GQ457566	GQ457562	GQ457547	GQ457556	GQ457553	GQ457550	GQ457559
	A/South Dakota/WRAIR1113P/2009	CY069370	CY069371	CY069372	CY069373	CY069374	CY069375	CY069376	CY069377
	A/Tennessee/UR06-0379/2007	CY037782	CY037781	CY037780	CY037775	CY037778	CY037777	CY037776	CY037779
	A/Texas/JMM/53/2012	CY135123	CY135122	CY135121	CY135116	CY135119	CY135118	CY135117	CY135120
	A/Texas/UR06-0503/2007	CY027890	CY027889	CY027888	CY027883	CY027886	CY027885	CY027884	CY027887
	A/Texas/UR06-0582/2007	CY026722	CY026721	CY026720	CY026715	CY026718	CY026717	CY026716	CY026719
	A/Texas/WRAIR1118P/2009	CY100809	CY100810	CY100811	CY100812	CY100813	CY100814	CY100815	CY100816
	A/Vermont/UR06-0511/2007	CY026626	CY026625	CY026624	CY026619	CY026622	CY026621	CY026620	CY026623
	A/Vermont/UR06-0573/2007	CY026578	CY026577	CY026576	CY026571	CY026574	CY026573	CY026572	CY026575
**Eurasian avian**	A/duck/Italy/281904/2006	FJ432777	FJ432776	FJ432775	FJ432770	FJ432773	FJ432772	FJ432771	FJ432774
	A/duck/Italy/69238/2007	FJ432761	FJ432760	FJ432759	FJ432754	FJ432757	FJ432756	FJ432755	FJ432758
	A/duck/Shimane/188/1999	CY091589	CY091590	CY091591	CY091592	CY091593	CY091594	CY091595	CY091596
	A/goose/Italy/296426/2003	FJ432785	FJ432784	FJ432783	FJ432778	FJ432781	FJ432780	FJ432779	FJ432782
	A/mallard/Bavaria/185-8/2008	HQ259221	HQ259222	HQ259223	HQ259224	HQ259225	HQ259226	HQ259227	HQ259228
	A/mallard/Marquenterre/Z237/1983	DQ864507	DQ864506	DQ864508	GU066779	DQ864509	GU066780	GU066781	GU066782
**Eurasian swine**	A/swine/Bieganow/1/2001	CY115870	CY115871	CY115872	CY115873	CY115874	CY115875	CY115876	CY115877
	A/swine/Chachoengsao/NIAH587/2005	AB434325	AB434326	AB434327	AB434328	AB434329	AB434330	AB434331	AB434332
	A/swine/Chonburi/NIAH9469/2004	AB434301	AB434302	AB434303	AB434304	AB434305	AB434306	AB434307	AB434308
	A/swine/Cotes d'Armor/1624/2002	CY116385	CY116386	CY116387	CY116388	CY116389	CY116390	CY116391	CY116392
	A/swine/England/004772/2001	CY115917	CY115918	JX843272	CY115919	CY115920	CY115921	CY115922	CY115923
	A/swine/England/101692/1997	CY115932	CY115933	CY115934	CY115935	CY115936	CY115937	CY115938	CY115939
	A/swine/England/1093/2005	CY115948	CY115949	CY115950	CY115951	CY115952	CY115953	CY115954	CY115955
	A/swine/England/1131/2004	CY115956	CY115957	CY115958	CY115959	CY115960	CY115961	CY115962	CY115963
	A/swine/England/117316/1986	CY115972	CY115973	CY115974	CY115975	CY115976	CY115977	CY115978	CY115979
	A/swine/England/167655/1997	CY116001	CY116002	CY116003	CY116004	CY116005	CY116006	CY116007	CY116008
	A/swine/England/17787/2000	CY116009	CY116010	CY116011	CY116012	CY116013	CY116014	CY116015	CY116016
	A/swine/England/17788/2000	CY116017	CY116018	CY116019	CY116020	CY116021	CY116022	CY116023	CY116024
	A/swine/England/193667/1998	CY116025	CY116026	CY116027	CY116028	CY116029	CY116030	CY116031	CY116032
	A/swine/England/195852/1992	CY116033	CY116034	CY116035	CY116036	CY116037	CY116038	CY116039	CY116040
	A/swine/England/252411/1992	CY116055	CY116056	CY116057	CY116058	CY116059	CY116060	CY116061	CY116062
	A/swine/England/254/2002	CY116063	CY116064	CY116065	CY116066	CY116067	CY116068	CY116069	CY116070
	A/swine/England/283902/1993	CY116087	CY116088	CY116089	CY116090	CY116091	CY116092	CY116093	CY116094
	A/swine/England/33780/2006	CY116108	CY116109	CY116110	CY116111	CY116112	CY116113	CY116114	CY116115
	A/swine/England/35320/1999	CY116116	CY116117	CY116118	CY116119	CY116120	CY116121	CY116122	CY116123
	A/swine/England/383/2005	CY116140	CY116141	CY116142	CY116143	CY116144	CY116145	CY116146	CY116147
	A/swine/England/39572/2001	CY116148	CY116149	CY116150	CY116151	CY116152	CY116153	CY116154	CY116155
	A/swine/England/410711/1994	CY116164	CY116165	CY116166	CY116167	CY116168	CY116169	CY116170	CY116171
	A/swine/England/453/2006	CY116203	CY116204	CY116205	CY116206	CY116207	CY116208	CY116209	CY116210
	A/swine/England/57610/1999	CY116219	CY116220	CY116221	CY116222	CY116223	CY116224	CY116225	CY116226
	A/swine/England/68327/1998	CY116273	CY116274	CY116275	CY116276	CY116277	CY116278	CY116279	CY116280
	A/swine/England/745769/1995	CY116288	CY116289	CY116290	CY116291	CY116292	CY116293	CY116294	CY116295
	A/swine/England/771270/1995	CY116296	CY116297	CY116298	CY116299	CY116300	CY116301	CY116302	CY116303
	A/swine/England/79851/1990	CY116304	CY116305	CY116306	CY116307	CY116308	CY116309	CY116310	CY116311
	A/swine/Finnistere/800/2004	CY116353	CY116354	CY116355	CY116356	CY116357	CY116358	CY116359	CY116360
	A/swine/Fujian/204/2007	FJ536816	FJ536817	FJ536815	FJ536810	FJ536813	FJ536812	FJ536811	FJ536814
	A/swine/Gent/112/2007	CY116423	CY116424	CY116425	CY116426	CY116427	CY116428	CY116429	CY116430

A/swine/Gent/132/2005	CY116431	CY116432	CY116433	CY116434	CY116435	CY116436	CY116437	CY116438
A/swine/Guangdong/103/2002	GQ422382	GQ422383	GQ422384	GQ422385	GQ422386	GQ422387	GQ422388	GQ422389
A/swine/Guangdong/1/2010	HM189301	HM189302	HM189303	HM189308	HM189305	HM189306	HM189307	HM189304
A/swine/Guangdong/1408/2010	JN375003	JN375045	JN375075	JN375122	JN375153	JN375189	JN375230	JN375261
A/swine/Guangdong/1613/2010	JN375019	JN375060	JN375096	JN375132	JN375163	JN375200	JN375235	JN375272
A/swine/Guangdong/34/2006	CY089864	CY089865	CY089866	CY089867	CY089868	CY089869	CY089870	CY089871
A/swine/Guangdong/L3/2009	HQ877024	HQ877025	HQ877026	HQ877027	HQ877028	HQ877029	HQ877030	HQ877031
A/swine/Guangxi/12/2005	HQ541663	HQ541662	HQ541661	HQ541656	HQ541659	HQ541658	HQ541657	HQ541660
A/swine/Heilongjiang/44/2009	HQ541694	HQ541695	HQ541693	HQ541688	HQ541691	HQ541690	HQ541689	HQ541692
A/swine/Hokkaido/2/1981	AB434389	AB434390	AB434391	AB434392	AB434393	AB434394	AB434395	AB434396
A/swine/Holguin/121/2010	HE584745	HE584746	HE584747	HE584748	HE584749	HE584750	HE584751	HE584752
A/swine/Hong Kong/1290/1993	CY085078	CY085079	CY085080	CY085081	CY085082	CY085083	CY085084	CY085085
A/swine/Hong Kong/1535/2009	CY061706	CY061707	CY061708	CY061709	CY061710	CY061711	CY061712	CY061713
A/swine/Hong Kong/1559/2008	CY085875	CY085876	CY085877	CY085878	CY085879	CY085880	CY085881	CY085882
A/swine/Hong Kong/227/2002	CY085491	CY085492	CY085493	CY085494	CY085495	CY085496	CY085497	CY085498
A/swine/Hong Kong/2433/2009	CY061818	CY061819	CY061820	CY061821	CY061822	CY061823	CY061824	CY061825
A/swine/Hong Kong/2445/1994	CY087069	CY087070	CY087071	CY087072	CY087073	CY087074	CY087075	CY087076
A/swine/Hong Kong/245/2009	CY085931	CY085932	CY085933	CY085934	CY085935	CY085936	CY085937	CY085938
A/swine/Hong Kong/2461/1994	CY087077	CY087078	CY087079	CY087080	CY087081	CY087082	CY087083	CY087084
A/swine/Hong Kong/3001/2009	CY061874	CY061875	CY061876	CY061877	CY061878	CY061879	CY061880	CY061881
A/swine/Hong Kong/347/1993	CY084990	CY084991	CY084992	CY084993	CY084994	CY084995	CY084996	CY084997
A/swine/Hong Kong/414/2009	CY085987	CY085988	CY085989	CY085990	CY085991	CY085992	CY085993	CY085994
A/swine/Hong Kong/435/2007	CY085827	CY085828	CY085829	CY085830	CY085831	CY085832	CY085833	CY085834
A/swine/Hong Kong/5683/1999	CY087133	CY087134	CY087135	CY087136	CY087137	CY087138	CY087139	CY087140
A/swine/Hong Kong/638/2003	CY085555	CY085556	CY085557	CY085558	CY085559	CY085560	CY085561	CY085562
A/swine/Hong Kong/676/2008	CY085851	CY085852	CY085853	CY085854	CY085855	CY085856	CY085857	CY085858
A/swine/Hong Kong/71/2009	CY085891	CY085892	CY085893	CY085894	CY085895	CY085896	CY085897	CY085898
A/swine/Hong Kong/7191/2000	CY085315	CY085316	CY085317	CY085318	CY085319	CY085320	CY085321	CY085322
A/swine/Hong Kong/72/2007	CY085811	CY085812	CY085813	CY085814	CY085815	CY085816	CY085817	CY085818
A/swine/Hong Kong/752/2002	CY085499	CY085500	CY085501	CY085502	CY085503	CY085504	CY085505	CY085506
A/swine/Hong Kong/7635/2000	CY085339	CY085340	CY085341	CY085342	CY085343	CY085344	CY085345	CY085346
A/swine/Hong Kong/7945/2000	CY085363	CY085364	CY085365	CY085366	CY085367	CY085368	CY085369	CY085370
A/swine/Hong Kong/8278/2001	CY085371	CY085372	CY085373	CY085374	CY085375	CY085376	CY085377	CY085378
A/swine/Hong Kong/835/1993	CY085062	CY085063	CY085064	CY085065	CY085066	CY085067	CY085068	CY085069
A/swine/Hong Kong/8631/2001	CY085387	CY085388	CY085389	CY085390	CY085391	CY085392	CY085393	CY085394
A/swine/Hong Kong/8709/2001	CY085403	CY085404	CY085405	CY085406	CY085407	CY085408	CY085409	CY085410
A/swine/Hong Kong/NS1022/2001	CY085427	CY085428	CY085429	CY085430	CY085431	CY085432	CY085433	CY085434
A/swine/Hong Kong/NS1071/2009	CY061682	CY061683	CY061684	CY061685	CY061686	CY061687	CY061688	CY061689
A/swine/Hong Kong/NS120/2007	CY085819	CY085820	CY085821	CY085822	CY085823	CY085824	CY085825	CY085826
A/swine/Hong Kong/NS129/2003	CY085547	CY085548	CY085549	CY085550	CY085551	CY085552	CY085553	CY085554
A/swine/Hong Kong/NS1344/2001	CY085451	CY085452	CY085453	CY085454	CY085455	CY085456	CY085457	CY085458
A/swine/Hong Kong/NS143/2000	CY085347	CY085348	CY085349	CY085350	CY085351	CY085352	CY085353	CY085354
A/swine/Hong Kong/NS1680/2001	CY085475	CY085476	CY085477	CY085478	CY085479	CY085480	CY085481	CY085482
A/swine/Hong Kong/NS184/2009	CY085971	CY085972	CY085973	CY085974	CY085975	CY085976	CY085977	CY085978
A/swine/Hong Kong/NS43/2009	CY085907	CY085908	CY085909	CY085910	CY085911	CY085912	CY085913	CY085914
A/swine/Hong Kong/NS613/2009	CY061626	CY061627	CY061628	CY061629	CY061630	CY061631	CY061632	CY061633
A/swine/Hong Kong/NS952/2008	CY085867	CY085868	CY085869	CY085870	CY085871	CY085872	CY085873	CY085874
A/swine/Hubei/02/2008	JX138514	JX138515	JX138516	JX138517	JX138518	JX138519	JX138520	JX138521
A/swine/Hubei/104/2009	CY091766	CY091767	CY091768	CY091769	CY091770	CY091771	CY091772	CY091773

	A/swine/Hunan/26/2010	HQ541655	HQ541654	HQ541653	HQ541649	HQ541652	HQ541651	HQ541650	HQ541648
	A/swine/Ille et Vilaine/1455/1999	CY116377	CY116378	CY116379	CY116380	CY116381	CY116382	CY116383	CY116384
	A/swine/Italy/1369-7/1994	CY098497	CY098498	CY098499	CY098500	CY098501	CY098502	CY098503	CY098504
	A/swine/Italy/1513-1/1998	CY116455	CY116456	CY116457	CY116458	CY116459	CY116460	CY116461	CY116462
	A/swine/Italy/151672-3/2003	CY116463	CY116464	CY116465	CY116466	CY116467	CY116468	CY116469	CY116470
	A/swine/Italy/172336/2001	CY116484	CY116485	CY116486	CY116487	CY116488	CY116489	CY116490	CY116491
	A/swine/Italy/186678-2/2005	CY116492	CY116493	CY116494	CY116495	CY116496	CY116497	CY116498	CY116499
	A/swine/Italy/29313/2006	CY116516	CY116517	CY116518	CY116519	CY116520	CY116521	CY116522	CY116523
	A/swine/Italy/670/1987	CY025260	CY025259	CY025258	CY025253	CY025256	CY025255	CY025254	CY025257
	A/swine/Jiangsu/s16/2011	JF820282	JF820283	JF820284	JF820285	JF820286	JF820287	JF820288	JF820289
	A/swine/Jiangsu/zg13/2011	JN809104	JN809122	JN809139	JN809159	JN809176	JN809193	JN809211	JN809230
	A/swine/Jiangsu/zg14/2011	JN809105	JN809123	JN809140	JN809160	JN809177	JN809194	JN809212	JN809231
	A/swine/Jiangsu/zg15/2011	JN809106	JN809124	JN809141	JN809161	JN809178	JN809199	JN809213	JN809232
	A/swine/Jiangsu/zg2/2010	JN809094	JN809112	JN809130	JN809148	JN809166	JN809184	JN809201	JN809220
	A/swine/Jiangsu/zg4/2010	JN809097	JN809115	JN809132	JN809150	JN809169	JN809187	JN809204	JN809223
	A/swine/Jiangsu/zg5/2010	JN809096	JN809114	JN809131	JN809151	JN809168	JN809186	JN809203	JN809222
	A/swine/Jiangsu/zg6/2010	JN809098	JN809116	JN809133	JN809152	JN809170	JN809188	JN809205	JN809224
	A/swine/Jiangsu/zg8/2010	JN809100	JN809118	JN809135	JN809154	JN809172	JN809190	JN809207	JN809226
	A/swine/Lutol/3/2000	CY116528	CY116529	CY116530	CY116531	CY116532	CY116533	CY116534	CY116535
	A/swine/Okinawa/1/2005	AB600242	AB600560	AB600844	AB600849	AB600854	AB600859	AB600864	AB600943
	A/swine/OMS/2111/1995	CY116393	CY116394	CY116395	CY116396	CY116397	CY116398	CY116399	CY116400
	A/swine/OMS/2112/1995	CY116401	CY116402	CY116403	CY116404	CY116405	CY116406	CY116407	CY116408
	A/swine/Ratchaburi/NIAH101942/2008	AB514933	AB514934	AB514935	AB514936	AB514939	AB514941	AB514942	AB514943
	A/swine/Ratchaburi/NIAH1481/2000	AB434285	AB434286	AB434287	AB434288	AB434289	AB434290	AB434291	AB434292
	A/swine/Ratchaburi/NIAH550/2003	AB434293	AB434294	AB434295	AB434296	AB434297	AB434298	AB434299	AB434300
	A/swine/Saraburi/NIAH100761-22/2009	AB620160	AB620161	AB620162	AB620163	AB620164	AB620165	AB620166	AB620167
	A/swine/Shandong/275/2009	GU086062	GU086063	GU086064	GU086065	GU086066	GU086067	GU086068	GU086069
	A/swine/Spain/50047/2003	CY009899	CY009898	CY009897	CY009892	CY009895	CY009894	CY009893	CY009896
	A/swine/Spain/51915/2003	CY010579	CY010578	CY010577	CY010572	CY010575	CY010574	CY010573	CY010576
	A/swine/Spain/53207/2004	CY010587	CY010586	CY010585	CY010580	CY010583	CY010582	CY010581	CY010584
	A/swine/Tianjin/01/2004	EU004447	EU004446	EU004445	EU004444	EU004443	EU004442	EU004440	EU004441
**Eurasian human**	A/Beijing/262/1995	CY033621	CY033620	CY033619	CY033614	CY033617	CY033616	CY033615	CY033618
	A/DaNang/DN238/2008	CY104885	CY104884	CY104883	CY104878	CY104881	CY104880	CY104879	CY104882
	A/England/493/2006	FJ445082	FJ445043	FJ445056	FJ445027	FJ445088	FJ445052	FJ445077	FJ445039
	A/England/593/2006	FJ445071	FJ445034	FJ445035	FJ445073	FJ445042	FJ445058	FJ445051	FJ445049
	A/England/594/2006	FJ445067	FJ445055	FJ445078	FJ445047	FJ445074	FJ445066	FJ445048	FJ445086
	A/Fujian/156/2000	CY125131	CY125130	CY125129	CY125124	CY125127	CY125126	CY125125	CY125128
	A/HaNoi/HN981/2003	CY104821	CY104820	CY104819	CY104814	CY104817	CY104816	CY104815	CY104818
	A/HaNoi/Q580/2006	CY104291	CY104290	CY104289	CY104284	CY104287	CY104286	CY104285	CY104288
	A/HoChiMinh/HCM554/2006	CY104669	CY104668	CY104667	CY104662	CY104665	CY104664	CY104663	CY104666
	A/Malaysia/12530/1997	CY117751	CY117750	CY117749	CY117744	CY117747	CY117746	CY117745	CY117748
	A/Malaysia/14075/1997	CY119105	CY119104	CY119103	CY119098	CY119101	CY119100	CY119099	CY119102
	A/Malaysia/14210/1997	CY119113	CY119112	CY119111	CY119106	CY119109	CY119108	CY119107	CY119110
	A/Malaysia/14817/1997	CY119121	CY119120	CY119119	CY119114	CY119117	CY119116	CY119115	CY119118
	A/Malaysia/1715991/2007	CY119241	CY119240	CY119239	CY119234	CY119237	CY119236	CY119235	CY119238
	A/Malaysia/1828663/2007	CY118106	CY118105	CY118104	CY118099	CY118102	CY118101	CY118100	CY118103
	A/Malaysia/33132/2005	CY119185	CY119184	CY119183	CY119178	CY119181	CY119180	CY119179	CY119182
	A/Malaysia/33166/2005	CY119193	CY119192	CY119191	CY119186	CY119189	CY119188	CY119187	CY119190
	A/Nanchang/19/1996	CY013852	CY013851	CY013850	CY013845	CY013848	CY013847	CY013846	CY013849

	A/Niigata/07F102/2008	CY043382	CY043383	CY043384	CY043385	CY043386	CY043387	CY043388	CY043389
	A/Philippines/WRAIR1736P/2006	CY100977	CY100978	CY100979	CY100980	CY100981	CY100982	CY100983	CY100984
	A/Russia/2187/2002	CY125171	CY125170	CY125169	CY125164	CY125167	CY125166	CY125165	CY125168
	A/Siena/10/1989	CY036830	CY036829	CY036828	CY036823	CY036826	CY036825	CY036824	CY036827
	A/Siena/4/1987	CY045859	CY045858	CY045857	CY045852	CY045855	CY045854	CY045853	CY045856
	A/Siena/9/1989	CY036950	CY036949	CY036948	CY036943	CY036946	CY036945	CY036944	CY036947
	A/St. Petersburg/8/2006	CY035133	CY035132	CY035131	CY035126	CY035129	CY035128	CY035127	CY035130
	A/Switzerland/5165/2010	CY079549	CY079542	CY079543	CY079544	CY079545	CY079546	CY079547	CY079548
	A/Taiwan/4845/1999	DQ415285	DQ415296	DQ415307	DQ415318	DQ415329	DQ415340	DQ415351	DQ415362
	A/Taiwan/567/2002	CY040081	CY040080	CY040079	CY040074	CY040077	CY040076	CY040075	CY040078
	A/TayNguyen/TN334/2005	CY105357	CY105356	CY105355	CY105350	CY105353	CY105352	CY105351	CY105354
	A/Thailand/CU44/2006	FJ912913	FJ912914	FJ912915	EU021258	FJ912916	EU021259	FJ912917	FJ912918
	A/TW/3355/1997	DQ415284	DQ415295	DQ415306	DQ415317	DQ415328	DQ415339	DQ415350	DQ415361
**2009 pandemic**	A/Boston/140/2009	CY064675	CY064674	CY064673	CY064668	CY064671	CY064670	CY064669	CY064672
	A/California/VRDL61/2009	CY055002	CY055001	CY055000	CY054995	CY054998	CY054997	CY054996	CY054999
	A/Guangdong/5301/2009	CY103903	CY103904	CY103905	CY103906	CY103907	CY103908	CY103909	CY103910
	A/Managua/3642.04/2009	CY072645	CY072644	CY072643	CY072638	CY072641	CY072640	CY072639	CY072642
	A/Mexico City/IBT23/2009	CY100453	CY100454	CY100455	CY100456	CY100457	CY100458	CY100459	CY100460
	A/New/York/5988/2009	CY056626	CY056625	CY056624	CY056619	CY056622	CY056621	CY056620	CY056623
	A/Quebec/144180/2009	FN434467	FN434466	FN434465	FN434469	FN434468	FN434470	FN434471	FN434472
	A/San Diego/INS62/2009	CY083676	CY083675	CY083674	CY083669	CY083672	CY083671	CY083670	CY083673
	A/Texas/JMS356/2009	CY060850	CY060849	CY060848	CY060843	CY060846	CY060845	CY060844	CY060847
	A/Wisconsin/629-D00859/2009	CY063306	CY063305	CY063304	CY063299	CY063302	CY063301	CY063300	CY063303

[a] The geography and host column was colored based on the taxa color in the phylogenetic trees shown in S1-S8 Figures.

[b] Pathogenicity index for North American avian viruses from St. Jude influenza repository was calculated as a function of surival and weight loss (Kocer et al., 2012)
