# Supplementary material for: The Genomic Contributions of Avian H1N1 Influenza A Viruses to the Evolution of Mammalian Strains
Source: PLoS One. 2015 Jul 24;10(7):e0133795. doi: 10.1371/journal.pone.0133795 (PMC4514870; doi:10.1371/journal.pone.0133795)
Supplement: S2 Table — (PDF) [file pone.0133795.s008.pdf]

S2 Table. Amino acid substitutions at the HA antigenic sites in avian, human*, and pandemic H1N1 IAVs

Year isolated	Antigenic Site	Amino Acid Position and Substitutions												
	Sa	141	142	170	171	172	173	174	176	177	178	179	180	181
1918		P	N	K	K	G	S	S	P	K	L	S	K	S
1934		P	N	E	K	E	G	S	P	K	L	K	N	S
1942		P	K	E	K	D	G	S	P	N	L	N	N	S
1950		P	K	E	K	N	G	S	P	N	L	K	K	S
1977		P	K	E	K	N	G	S	P	N	L	S	K	S
1986		P	N	K	K	N	G	S	P	N	L	S	K	S
1991		P	N	K	K	N	G	L	P	N	V	S	K	S
1998		P	N	E	K	N	G	L	P	N	L	S	K	S
2007		P	N	G	K	N	G	L	P	N	L	S	K	S
2009		P	N	K	K	G	N	S	P	K	L	S	K	S
**Char**		P	N	K	K	G	N	S	P	K	I	S	K	S
**Anser**		P	N	K	K	G	T/N	S	P	K	L	S	K	S

Ca	154	155	156	157	158†	159	183	184	185	186	187	220	221	222	238	239	252	253	254
1918	S	Y	A	G	A	S	V	N	N	K	G	S	S	K	R	D	E	P	G
1934	S	H	E	G	K	S	V	N	K	K	G	T	S	N	R	D	K	P	G
1942	S	H	A	G	K	C	V	N	K	K	G	S	S	N	R	G	E	P	G
1950	S	H	K	G	K	S	V	N	N	K	E	S	S	N	R	G	E	P	G
1977	S	H	K	G	K	S	V	N	N	K	E	S	S	N	R	G	E	P	G
1986	S	H	K	G	R	S	V	N	N	K	E	S	S	H	R	D	E	P	G
1991	S	H	N	G	K	S	V	N	N	K	E	S	S	H	R	D	E	P	G
1998	S	H	N	G	K	S	V	N	N	K	E	S	S	H	R	D	E	P	G
2007	S	H	N	G	E	S	A	N	N	K	E	S	S	H	R	D	E	P	G
2009	P	H	A	G	A	K	I	N	D	K	G	S	S	R	R	D	E	P	G
Char	P	Y	S	G	V	S	T	N	N	K	G	S	S	K	R	G	D	Q	G
Anser	S	Y	S	G	A	S	T	N	N	K	G	S	S	K	R	G	D	Q	G

	Sb	201	202	203	204	205	206	207	208	209	210	211	212†
1918		T	G	T	D	Q	Q	S	L	Y	Q	N	A
1934		N	S	K	E	Q	Q	N	I	Y	Q	N	E
1942		N	I	K	D	Q	Q	T	L	Y	Q	K	E
1950		N	I	E	D	Q	K	T	L	Y	R	K	E
1977		N	I	E	D	Q	K	T	I	Y	R	K	E
1986		N	I	G	D	Q	R	A	I	Y	H	T	E
1991		N	I	G	D	Q	R	A	I	Y	H	T	E
1998		N	I	G	D	Q	R	A	I	Y	H	T	E
2007		N	I	G	D	Q	K	A	L	Y	H	T	E
2009		T	S	A	D	Q	Q	S	L	Y	Q	N	A
**Char**		T	T	S	E	Q	Q	S	L	Y	Q	N	I
**Anser**		T	T	S/N	E	Q	Q	S	L	Y	Q	N	T/A

Cb	87	88	89	90	91	92
1918	L	L	T	A	S	S
1934	L	L	P	V	R	S
1942	L	L	S	E	R	S
1950	L	F	S	K	K	S
1977	L	F	S	K	K	S
1986	L	F	S	K	K	S
1991	L	F	S	K	E	S
1998	L	I	S	K	E	S
2007	L	I	S	K	E	S
2009	L	S	T	A	S	S
**Char**	L	L	T	A	N	S
**Anser**	L	L	T	A	N	S

fully conserved
largely conserved#
1918 & 2009 avian-like
1918-like only
2009-like only
avian-like

*The antigenic sites seen in human viruses between 1918 and 2009 were acquired from Igarashi et al [24].

†Positions 158 in Ca and 212 in Sb are "pandemic-like only" in the H1N1 IAVs of Anseriformes origin but not in those of Charadriiformes origin (shown in light blue).

#Residues were considered largely conserved when the same amino acid residue observed in many isolates over the years.
